# Environmental impacts of shared mobility: a systematic literature review of life-cycle assessments focusing on car sharing, carpooling, bikesharing, scooters and moped sharing

**DOI:** 10.1080/01441647.2023.2259104

**Published:** 2023-11-13

**Authors:** Ana María Arbeláez Vélez

**Affiliations:** International Institute of Industrial Environmental Economics, Lund University, Lund, Sweden

**Keywords:** Shared mobility, life-cycle assessment, environmental impacts, car sharing, micromobility sharing

## Abstract

Evidence about the environmental impacts of shared mobility is fragmented and scattered. In this article a systematic literature review is presented. The review focuses on assessments that use Life-Cycle Assessment to quantify the environmental impacts of car sharing, carpooling, bikesharing, and scooter/moped sharing. The results of these assessments were analyzed, as well as the factors that influence these impacts. Business-to-consumer car sharing, peer-to-peer car sharing, carpooling, bikesharing, and scooter/moped sharing can all cause gains and losses in terms of changing the environmental impacts of passenger transportation. The findings presented here refute unconditional claims that shared mobility delivers environmental benefits. Factors that influence changes in environmental impacts from passenger transportation from shared mobility include travel behaviour, the design of shared mobility modes, and how such schemes are implemented, as well as the local context. Local governments and shared mobility organisations can benefit from the analysis presented here by deepening their understanding of these factors and considering the life-cycle phase where the greatest impacts are caused.

## 1. Introduction

Greenhouse gas (GHG) emissions from passenger transportation continue to grow, despite improvements in energy efficiency in this sector (Lamb et al., 2021; Sims et al., 2014a). This rise in emissions is related to increasing transportation activity and a switch to more emission-intensive transportation modes (Lamb et al., 2021; Sims et al., 2014b). Passenger transportation is linked to other impacts including noise, material resource depletion, and land use.

Shared mobility is one demand-side solution that has the potential to change how people travel (Shaheen & Cohen, 2018a). Shared mobility provides customers with short-term access to vehicles or transportation services in exchange for a fee or for free (Shaheen et al., 2016). In addition to older forms such as carpooling, many new shared mobility modes have emerged in the last two decades with the advent of the Internet, including business-to-consumer (B2C) car sharing, peer-to-peer (P2P) car sharing, bikesharing, and scooter/moped sharing (Shaheen et al., 2016).

The extent to which these transportation modes deliver environmental gains is debated, as environmental assessments of shared mobility show mixed results (Shaheen & Cohen, 2018a). Some studies claim shared mobility has positive environmental impacts (Amatuni et al., 2020; Lausselet & Brattebø, 2021; Martin & Shaheen, 2011; Severis et al., 2019), while others conclude the opposite or show mix results (Arbeláez Vélez & Plepys, 2021; de Bortoli, 2021; de Bortoli & Christoforou, 2020; Ding et al., 2019; Hollingsworth et al., 2019). The spotty and inconsistent picture regarding the environmental impacts of shared mobility makes it difficult to design policies that harness its positives and dampen its negative impacts (Shaheen & Cohen, 2018b). Moreover, the factors that influence these impacts are unclear.

Here these gaps are addressed through a systematic literature review focusing on studies that quantify the environmental impacts of car sharing, carpooling, bikesharing, and scooter or moped sharing schemes, concentrating on the results of their assessments and the factors that influence them. This review compiled studies that use Life-Cycle Assessment (LCA) to evaluate environmental impacts. LCA is a method that can calculate a variety of environmental impacts based on an extensive inventory of materials and energy used throughout the life phases of a product or service, encompassing raw material extraction, production, use, end-of-life (EoL), and transportation infrastructure building (International Organization for Standardization, 2006).

Through this review, this article makes two contributions to the literature: (1) it compiles and systematically reviews the existing literature regarding the environmental impacts of shared mobility modes, and (2) it distills the factors that influence these environmental impacts. In this review we discuss how shared mobility has the potential to both exacerbate and dampen environmental impacts of passenger transportation depending on some factors that were identified through a systematic literature review.

## 2. Background

Shared mobility modes differ in terms of their characteristics, including the type of vehicle shared (e.g. bikes, cars, or scooters), the form of ownership (either privately owned as in P2P schemes or corporate-owned as in B2C schemes), how shared vehicles are parked (free-floating or at fixed stations), and various specifics in terms of liability and pricing (Curtis & Lehner, 2019). Stationary schemes refer to vehicle-sharing systems where the vehicle is picked up and returned to designated parking locations, while free-floating refers to sharing systems where users can pick up and return vehicles to any appropriate location within a designated area. In the case of bike, scooter, and moped sharing systems, stationary systems are referred to as docked while free-floating systems are referred to as dockless.

Table 1 includes a short description of the shared mobility modes included in this review, specifying the parking type, ownership type, and classification, and providing examples of each designation. The system for classifying shared mobility modes laid out in this table will be used throughout the rest of this article. Passenger car sharing refers to shared mobility modes that use cars, i.e. car sharing or carpooling. Micromobility refers to systems for sharing bikes, scooters, or mopeds.

**Table 1.** Classification system for the shared mobility modes included in this review.

Name	Description	Parking type	Ownership type	Classification	Examples^1^
Car sharing^2^ or B2C car sharing.	Users can access cars located around the city. They pay a either a flat fee or per-usage rate (per hour, day or kilometer traveled)	Stationary-based (round-trip, one-way)	Business owned	Passenger car sharing	Enterprise, Greenwheels
		Free-floating (one-way)	Business owned	Passenger car sharing	Share Now, Zipcar, Communauto
P2P car sharing or personal vehicle sharing^2^	Car owners share their car with others when they are not using it. In exchange they can receive monetary compensation.	Stationary-based (round-trip)	Privately owned	Passenger car sharing	GoMore, Turo
Carpooling^2^	Car owners share available space in their cars with individuals with similar travel routes or destinations. For this, they receive monetary compensation	P2P Carpooling	Privately owned	Passenger car sharing	BlaBlaCar, Waze Carpool, Poparide, TooGethr, DiDi
	Companies provide employees with a car to commute together in	B2C Carpooling	Business owned	Passenger car sharing	Enterprise, Smart Commute
Bikesharing^2^	Bikes are available around the city. Several people can access the bike but not simultaneously. Users might pay a fixed fee or per-usage rate	Dockless, docked and hybrid systems	Business, government or private-public partnership owned	Micromobility sharing	BLOOM, Oslo Bysykkel, OV-fiets
Scooter sharing	E-scooters are available around the city. Several people can access the e-scooters but not simultaneously. Users pay per usage	Dockless, docked and hybrid systems	Business owned.	Micromobility sharing.	GrabWheels, Voi, TIER
Moped sharing	Moped sharing systems work similar to e-scooters, with the main difference being the vehicle used which can be a moped or e-moped	Dockless	Business owned	Micromobility sharing	Felyx, Coup, emmy

^1^ These examples might be out of date by the time this literature review is published, given the fast-changing landscape of shared mobility companies.

^2^ Shaheen et al. (2016).

## 2. Method

The systematic literature review followed the ROSES methodology (Haddaway et al., 2017). The ROSES methodology offers a structured process to plan and develop systematic literature reviews within the field of conservation and environmental management. This methodology provides guidelines, including flow diagrams, check lists, and templates, to document each step of the process in a consistent manner.

First, the search strategy will be explained, followed by the inclusion criteria for selecting studies and the process for extracting data.

### 2.1. Search strategy

The search was conducted using the Scopus and Web of Science databases in March 2021, followed by an update in October 2021. The search looked at titles, keywords, and abstracts and was limited to peer-reviewed articles. Two substrings were part of the search string (Table 2). The first one – shared mobility (X) – captures the shared mobility options to be researched. This string was based on literature reviews that made an inventory of shared mobility modes (Machado et al., 2018; Shaheen et al., 2016). The second substring – assessments (Y) – was based on a string used in a previous study with a similar focus (Ivanova et al., 2020). These two substrings were connected to form the full search string: X AND Y.

**Table 2.** Substrings used in the systematic search^1^.

Substring X: Shared mobility	(“shared mobility”) OR (carsharing) OR (“car shar*”) OR (“bike shar*”) OR (bikeshar*) OR (escooter*) OR (“ride shar*”) OR (rideshar*) OR (micromobility) OR (ridesourcing) OR (ridesplitting) OR (e-scooter*) OR (carpool*)
Substring Y: Assessments	(“Life-cycle a*”) OR (“environmental evaluation”) OR (“Environmental impact”) OR (emissions) OR (“sustainability impacts”) OR (“urban impacts”) OR (“environmental assessment”) OR (“sustainability”) OR (“environmental performance”) OR (carbon)

^1^ * Is used in search strings to retrieve words that start in the same way but end differently. For example, car shar* retrieves car-sharing or car share.

### 2.2. Study selection criteria

**Figure 1.**
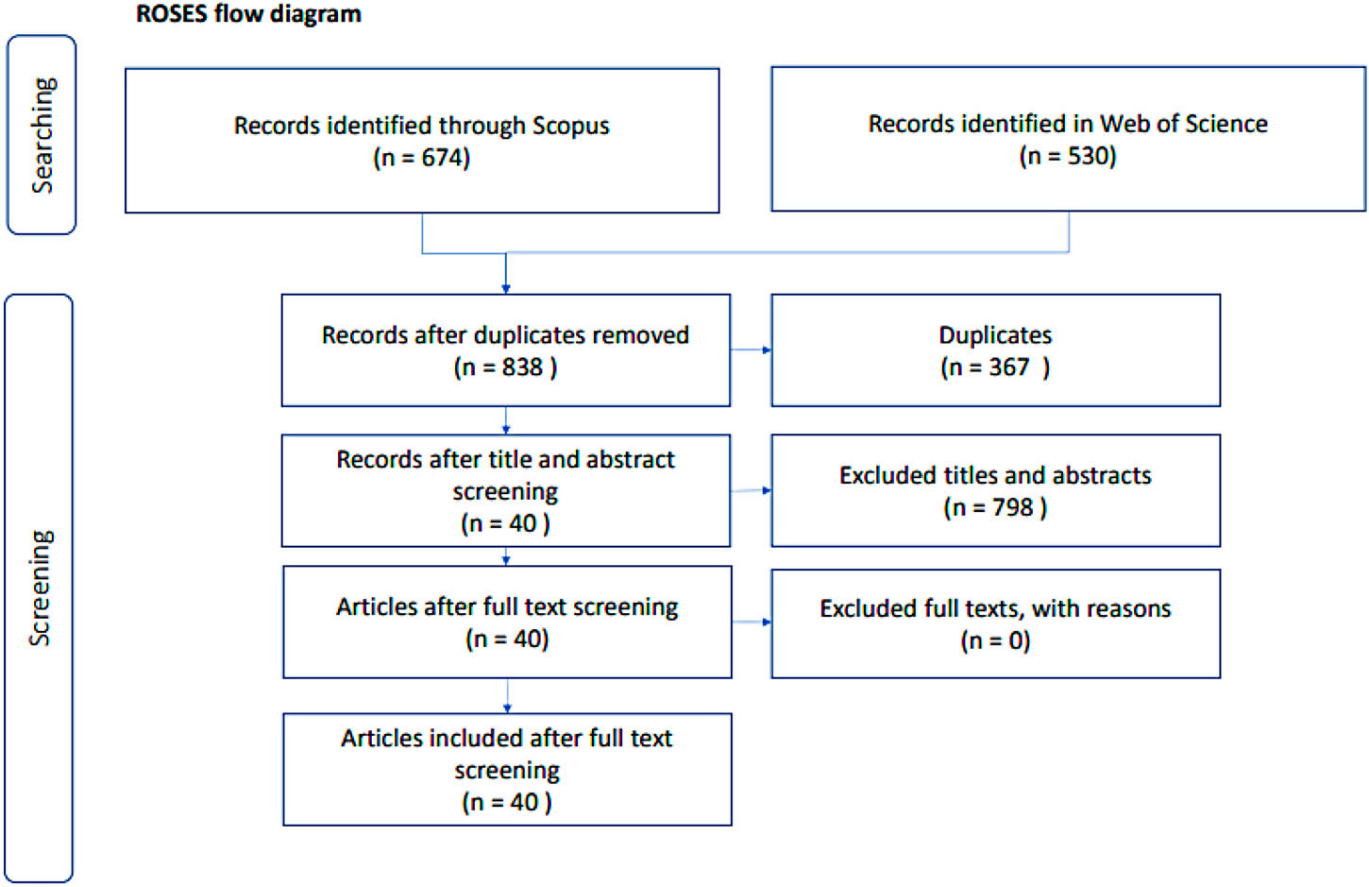
ROSES flow diagram.

Figure 1 shows the process of selecting the articles that were included in this literature review. First, the results from both search engines were filtered and duplicates were eliminated, leaving 838 articles. The first screening focused on the titles and abstracts, resulting in 40 articles to be fully reviewed.

Articles assessing car sharing, carpooling, bikesharing, and scooters or moped sharing were included. Ride-hailing was not included in this review, given that other literature reviews have already focused on this form of mobility (Chalermpong et al., 2022; Greenblatt & Shaheen, 2015; Khavarian-Garmsir et al., 2021; Tirachini, 2020). This article focuses on quantitative assessments that use LCA, given that this method can encompass the whole lifecycle of the transportation service and takes a holistic perspective (European Commission, 2021). LCA is a method that explores impacts beyond the use phase and is capable of identifying possible trade-offs among impact categories (European Commission, 2021). One example would be a specific transportation mode that performs well in carbon dioxide (CO_2_) emissions but requires significant use of material resources.

This study is confined to academic literature published in English after 2006 and thus omits studies in other languages. The resulting samples include cases from North America, Europe, Asia, and Latin America, offering a diversity of urban contexts. Studies were not excluded based on the location they assessed. For details about the studies included in this review see Annex 1 in Supplementary material.

### 2.2. Data extraction and coding

A single coder coded and analyzed the extracted data using the Gioia method, a three-step qualitative method to systematically review large amounts of data (Gioia et al., 2013). The first step is coding the data and establishing first-order categories. This step is inductive, which means that the data informs the emerging codes. For example, in the reviewed article of Zhang and Mi (2018) they specified that they quantified CO_2_ and nitrogen oxides (NO_x_) emissions, along with fuel consumption. In these first-order categories, the researcher documented these impacts in a spreadsheet as “CO_2_ emissions”, “NO_x_ emissions”, and “fuel consumption”, In the second step, the coder reviews the first-order categories and groups them into second-order themes. In Zhang and Mi (2018) second-order themes grouped *CO_2_ emissions* and *NO_x_ emissions* into the theme *climate change*, while *fuel consumption* was grouped into *resource depletion*. In this study, these two steps were used to code the environmental impact categories and level of analysis (see Annex 2 in Supplementary material for information regarding the environmental impacts evaluated and their classification).

The third step in the Gioia method was used to identify the factors that influenced environmental impacts. In this step, the coder reviewed the articles, extracting the variables used to quantify environmental impacts as specified in the methods or results section. Some of the reviewed articles included a sensitivity analysis, which we used here to inform the discussion of the relevance of the different variables. The third step in the Gioia method involves grouping the second-order themes in aggregate dimensions. The first-order categories, second-order themes and aggregate dimensions provide the basis for analysis of the data in the reviewed articles (Gioia et al., 2013). To facilitate the analysis, environmental impacts were grouped as shown in Table 3.

**Table 3.** Environmental impact categories and details about the impacts included.

Environmental impact category	Indicators
Air pollution	Fine particulate matter (PM_2.5_ and PM_10_) and emissions of sulfur dioxide (SO_2_), nitric oxide (NO) and NO_x_
Climate impacts	Emissions of CO_2_, methane (CH_4_)_,_ GHG, carbon monoxide (CO), global warming potential (GWP), and climate change
Ecosystem damage	Ecosystem damage, eutrophication potential, acidification potential, freshwater eutrophication, and terrestrial acidification
Indicator	Eco points and total normalised environmental impacts
Land use	Land use (parking area)
Ozone creation	Photochemical ozone creation potential
Resource depletion	Material depletion, energy consumption, mineral resource scarcity, fossil resource scarcity, energy consumption, fuel use, primary energy, abiotic depletion potential (ADP) water, ADP minerals, and fossils

## 3. Results

Table 4 presents the results of the car sharing assessments reviewed, Table 5 for carpooling, Table 6 for bikesharing, Table 7 for shared scooters, and Table 8 for shared mopeds and general shared micromobility systems. Each of these tables contains the results from the reviewed articles, specifying which environmental impacts were assessed and at which level the assessment was done. In this section, these results were analyzed, as well as the factors that influenced them. At the end of this section a summary of the factors that influenced the environmental impacts is presented (Table 9).

**Table 4.** Summary of environmental impacts from car sharing.

Environmental impact category	Indicators	Results	Level of analysis	Reference
Air pollution	NOx emissions	Private driving: 0.077 t Car sharing: 0.0892 t	City	Migliore et al. (2020)
	PM_10_	Private driving: 0.028 t Car sharing: 0.021 t	City	Migliore et al. (2020)
Climate impacts	CO_2_ emissions	15% reduction	Neighborhood	Lausselet et al. (2021)
		Private driving: 334.5 t Car sharing: 208.93 t	City	Migliore et al. (2020)
		35-65% reduction	City	Baptista et al. (2014)
	GHG emissions	Before car sharing: 0.00024 t CO_2_eq After low-use scenario: 0.00016 t CO_2_eq After medium-use scenario: 0.00012 t CO_2_eq After high-use scenario: 0.00007 t CO_2_eq	Per kilometer	Chen and Kockelman (2016)
		Car sharing (free-floating): 0.00024–0.00028 t CO_2_eq Car sharing (stationary): 0.00017–0.00019 t CO_2_eq Private car: 0.00025 t CO_2_eq Carpooling: 0.00020–0.00022 t CO_2_eq	Per kilometer	Sun and Ertz (2021b)
		Netherlands: 0.15–0.29 t CO2eq reduction San Francisco: 0.44–0.50 t CO2eq reduction Calgary: 0.084 reduction	Per person annual transportation	Amatuni et al. (2020)
		Increase of 0.025–0.023 t CO2eq or reduction of 0.92–0.94 t CO_2_eq	Per person annual transportation	Arbeláez Vélez and Plepys (2021)
		Best-case reduction: 0.31 t CO_2_eq Worse-case reduction: 0.15 t CO_2_eq	Per personal annual transportation	Firnkorn and Müller (2011)
		Increase of 0–0.25 t CO2eq or decrease of 0.50–0.65 t CO_2_eq	Per household annual transportation	Martin and Shaheen (2011)
		Decrease of 48%–55%	Per household annual transportation	Namazu and Dowlatabadi (2015)
		Savings of 136000 t CO_2_eq	Country	Te and Lianghua (2020)
	GWP	Private car: 3.60 t CO_2_eq per year Two nodes^1^: 2.24 t CO_2_eq per year Free-floating: 4.00 t CO_2_eq per year AB mode^1^: 4.54 t CO_2_eq per year Carpooling: 3.58 t CO_2_eq per year	Per vehicle lifetime	Ding et al. (2019)
	CH_4_	Private driving: 0.0258 t Car sharing: 0.0688 t	City	Migliore et al. (2020)
	CO	Private driving: 0.7309 t Car sharing: 0.7309 t	City	Migliore et al. (2020)
Land use	Land use	4.68 × 10^9 ^m^2^ reduction	Country	Te and Lianghua (2020)
Ozone depletion	Ozone depletion	Private driving: 0.1751 t Car sharing: 0.0291 t	City	Migliore et al. (2020)
Resource depletion	Energy use	Current shared system: No reduction Scenario with 3000 cars: 1853 t fuel savings	City	Zhang et al. (2021)
		Current shared system: No savings Scenario with 3000 cars: 3.36 GWh increase in energy consumption		
	Energy use	Before car sharing: 3.21 MJ After low-use scenario: 2.15 MJ After medium-use scenario: 1.55 MJ After high-use scenario: 0.98 MJ	Per kilometer	Chen and Kockelman (2016)
		35%–47% reduction	City	Baptista et al. (2014)
		1.67 × 10^9 ^MJ reduction	Country	Te and Lianghua (2020)

^1^ Two nodes: pick-up and drop the shared car in specific parking spot. AB mode: Pick-up at A and return at B or other designated parking stations.

**Table 5.** Summary of environmental impacts from car pooling.

Environmental impact category	Indicators	Results	Level of analysis	Reference
Air pollution	Primary PM_2.5_	Beijing: 306.9 t reduction Tianjin: 9.2 t reduction Shijiazhuang: 9.7 t reduction	City	Ma et al. (2018)
		Beijing: 306.9 t reduction	City	Yu et al. (2017)
	SO_2_ emissions	Beijing: 1149.2 t reduction Tianjin: 34.5 t reduction Shijiazhuang: 36.5 t reduction	City	Ma et al. (2018)
		Beijing: 1149.2 t reduction	City	Yu et al. (2017)
	NO_X_ emissions	Beijing: 1447 t reduction Tianjin: 43.5 t reduction Shijiazhuang: 36.7 t reduction	City	Ma et al. (2018)
		Beijing: 1447 t reduction	City	Yu et al. (2017)
Climate impacts	CO_2_ emissions	Beijing: 612.8 × 10^3^ t reduction Tianjin: 18.4 × 10^3^ t reduction Shijiazhuang: 19.5 × 10^3^ t reduction	City	Ma et al. (2018)
		Beijing: 612.8 × 10^3^ t reduction	City	Yu et al. (2017)
		8.96–29.97 t reduction	City	Farrell et al. (2010)
		17897 t reduction	Region	Stewart (2015)
		17.6 t reduction	Region	Buzzoni (2013)
		Scenario 1: 12674 t Scenario 2: 10139 t Scenario 3: 7604 t	Country	Caulfield (2009)
	GWP	Carpooling: 0.00020–0.00022 t CO_2_eq Car sharing (free-floating): 0.00024–0.00028 t CO_2_eq Car sharing (stationary): 0.00017–0.00019 t CO_2_eq Private car: 0.00025 t CO_2_eq	Per kilometer	Sun and Ertz (2021b)
		95 t CO_2_eq reduction	Neighborhood	Lausselet and Brattebø (2021)
Resource depletion	Energy use	Beijing: 196 × 10^3^ tce reduction	City	Yu et al. (2017)
	Material depletion	64 t Fe-eq reduction	Neighborhood	Lausselet and Brattebø (2021)
Ecosystem damage	Freshwater Eutrophication	83 t *P*-eq reduction	Neighborhood	Lausselet and Brattebø (2021)
	Terrestrial acidification	0.61 t SO_2_-eq reduction	Neighborhood	Lausselet and Brattebø (2021)
Index	Single score^1^	Private car: 49190 One extra person: 24590 Two extra people: 16400	Person	Severis et al. (2019)

^1^ Index that groups climate change, human toxicity, photo-chemical oxidant formation, particulate matter formation, terrestrial acidification, freshwater eutrophication, agricultural land occupation, metal depletion, fossil depletion and other midpoint categories.

**Table 6.** Summary of environmental impacts of bike sharing.

Environmental category	Indicators	Results	Level of analysis	Reference
Air pollution	NO_X_ emissions	64 t reduction	City	Zhang and Mi (2018)
Climate impacts	CO_2_ emissions	Bike sharing: 2.97 × 10^−6^ t Bus system: 1.37 × 10^−4^ t	Per trip	Wang et al. (2021)
		9.23 × 10^6^–9.26 × 10^6^ t reduction	City	Ding et al. (2021)
		25 240 t reduction	City	Zhang and Mi (2018)
		716–4300 t reduction	City	(D’Almeida et al. (2021)
	GHG emissions	Current: 1 × 10^−4^ t CO_2_eq Scenario 1: 5.5 × 10^−5^ t CO_2_eq Scenario 2: 6.4 × 10^−5^ t CO_2_eq Scenario 3: 8.0 × 10^−5^ t CO_2_eq	Per kilometer	Luo et al. (2020)
		Private: 7.47 × 10^−6^ t CO_2_eq Smart dockless: 1.29 × 10^−4^ t CO_2_eq Smart docked: 6.83 × 10^−5^ t CO_2_eq	Per kilometer	Bonilla-Alicea et al. (2020)
		Stationary-based system: 6.5 × 10^−5^ t CO_2_eq Free-floating: 1.18 × 10^−4^ t CO_2_eq Private bike: 1.0 × 10^−5^ t CO_2_eq Car: 2.06 × 10^−4^ t CO_2_eq Bus: 1.08 × 10^−4^ t CO_2_eq	Per kilometer	Luo et al. (2019)
		Private bike: 10.5 × 10^−6^ t CO_2_eq Station-based: 57.35 × 10^−6^–68.99 × 10^−6^ t CO_2_eq	Per kilometer	Sun and Ertz (2021b)
		116739 t CO_2_eq reduction	City	Ding et al. (2021)
	GWP	Bike sharing (high-low): Savings 13.42–23.86 g CO_2_eq Private bike: 4.57 g CO_2_eq Bus: 32.21 g CO_2_eq Private car: 151.53 g CO_2_eq	Per kilometer	Tao and Zhou (2021)
Indicator	Total normalised environmental impacts (unit)	Stationary-based system: 2.30 Free-floating: 1.49 Private bike: 0.82 Car: 4.20 Bus: 0.77	Per kilometer	Luo et al. (2019)
Resource depletion	Material depletion	Stationary: Al: 9 g/Steel: 29 g/Plastic: 1.56 g/Rubber: 0.9 g Free-floating: Al: 5.6 g/Steel: 6 g/Plastic: 2.1 g/Rubber: 1 g Private: Al: 9.9 g/Steel: 7.5 g/Plastic: 2.6 g/Rubber: 1.6 g	Per trip	Sun and Ertz (2021a)
	ADP minerals and fossils	Bike sharing (high-low): 0.40 × 10^−6^–3.32 × 10^−6 ^kg Sb.-eq Private bike: 0.98 × 10^−6^ kg Sb.-eq Bus: 0.4 × 10^−6 ^kg Sb.-eq Private car: 5.63× 10^−6 ^kg Sb.-eq	Per kilometer	Tao and Zhou (2021)
	ADP water	Bike sharing (high-low): 0.003–0.0349 g water-eq Private bike: 0.0160 g water-eq Bus: 0.0068 g water-eq Private car: 0.0789 g water-eq	Per kilometer	Tao and Zhou (2021)
	Energy consumption	Bike sharing: 0.0968 kJ Bus system: 1.27 kJ	Per *p*-mile	Wang et al. (2021)
	Fuel use	8358 t reduction	City	Zhang and Mi (2018)

**Table 7.** Summary of environmental impacts from e-scooter sharing.

Environmental category	Impacts	Results	Level of analysis	Reference
Air pollution	Fine particulate matter	Base case: 2.96 × 10^−4 ^kg PM_2.5_eq Scenario 1: 1.44 × 10^−4 ^kg PM_2.5_eq Scenario 2: 1.99 × 10^−4 ^kg PM_2.5_eq Scenario 3: 1.01 × 10^−4 ^kg PM_2.5_eq Scenario 4: 7.5 × 10^−4 ^kg PM_2.5_eq	Per kilometer	Moreau et al. (2020)
Climate impacts	CO_2_ emissions	49–300 g	Per kilometer	Kazmaier et al. (2020)
		Car: 209 g Private scooters: 135 g Bus: 133 g Shared bikes: 59 g Shared e-scooters: 109 g Metro: 8 g	Per kilometer	de Bortoli and Christoforou (2020)
		Base case: 131 g Scenario 1: 110 g Scenario 2: 91 g Scenario 3: 51 g Scenario 4: 40 g	Per kilometer	Moreau et al. (2020)
		E-scooters: 150 g Private car: 414 g Dockless bikes: 190 g Mopeds: 119 g Buses with high ridership: 82 g Electric bikes: 40 g Bikes: 8 g	Per mile	Hollingsworth et al. (2019)
		Base case: 178 × 10^3 ^g Scenario 1: 123 × 10^3 ^g Scenario 2: 134 × 10^3 ^g Scenario 3: 150 × 10^3 ^g Scenario 4: 152 × 10^3 ^g	Per shared vehicle	Hollingsworth et al. (2019)
Resource scarcity	Mineral resource scarcity	Base case: 1.97 × 10^−3 ^kg Cu-eq Scenario 1: 5.76 × 10^−4 ^kg Cu-eq Scenario 2: 1.30 × 10^−3 ^kg Cu-eq Scenario 3: 6.24 × 10^−4 ^kg Cu-eq Scenario 4: 4.66 × 10^−5 ^kg Cu-eq	Per kilometer	Moreau et al. (2020)
	Fossil resource scarcity	Base case: 3.16 × 10^−2 ^kg oil-eq Scenario 1: 3.43 × 10^−2 ^kg oil-eq Scenario 2: 2.26 × 10^−2 ^kg oil-eq Scenario 3: 1.36 × 10^−2 ^kg oil-eq Scenario 4: 1.12 × 10^−2 ^kg oil-eq	Per kilometer	Moreau et al. (2020)
Ecosystem damage	Acidification	Base case: 0.989^ ^kg SO_2_eq Scenario 1: 0.814^ ^kg SO_2_eq Scenario 2: 0.841^ ^kg SO_2_eq Scenario 3: 0.961^ ^kg SO_2_eq Scenario 4: 0.707^ ^kg SO_2_eq	Per shared vehicle	Hollingsworth et al. (2019)
	Eutrophication	Base case: 0.697^ ^kg Neq Scenario 1: 0.567^ ^kg Neq Scenario 2: 0.601^ ^kg Neq Scenario 3: 0.690^ ^kg Neq Scenario 4: 0.473^ ^kg Neq	Per shared vehicle	Hollingsworth et al. (2019)

**Table 8.** Summary of environmental impacts from moped sharing and other studies.

Environmental category	Impact	Results	Level of analysis	Shared mobility mode	Reference
Air pollution	SO_2_ emissions	Scenario 1: Current 0.166/Renewable 0.147 g SO_2_eq Scenario 2: Current 0.1712/Renewable 0.154 g SO_2_eq Scenario 3: Current 0.217/Renewable 0.198 g SO_2_eq	Per kilometer	Moped	Wortmann et al. (2021)
	PM_2.5_	Scenario 1: Current 0.062/Renewable 0.058 g PM_2.5_eq Scenario 2: Current 0.064/Renewable 0.06 g PM_2.5_eq Scenario 3: Current 0.081/Renewable 0.077 g PM_2.5_eq	Per kilometer	Moped	(Wortmann et al., 2021)
Climate impacts	CO_2_ emissions	Scenario 1: Current 32.3 g/Renewable 18.7 g Scenario 2: Current 32.9 g/Renewable 19.3 g Scenario 3: Current 36.7 g/Renewable 23.1 g	Per kilometer	Moped	Wortmann et al. (2021)
		Moped sharing: 20–58 g Electric buses: 27–52 g Bicycle: 10sg Bus: 80 g Moped short service life: 58 g Moped less rebalancing: 25 g Moped solar powered charging: 41 g Moped less rebalancing & solar powered charging: 20 g	Per kilometer	Moped	Schelte et al. (2021)
		Shared bike: 32.9 g Private bike: 11.7 g Shared e-scooter: 61.0 g Private e-scooter: 59.5 g Shared moped: 34.0 g Private motorcycle: 14.3 g	Per kilometer	Micromobility sharing	de Bortoli (2021)
Resource depletion	Energy consumption	Shared bike: 1040 kJeq Private bike: 159 kJeq Shared e-scooter: 1310 kJeq Private e-scooter: 938−1150 kJeq Shared moped: 1200 kJeq Private motorcycle: 2490 kJeq	Per kilometer	Micromobility sharing	de Bortoli (2021)
		Scenario 1: Current – 531.9 kJeq Renewable – 400.6 kJeq Scenario 2: Current – 539.3 kJeq Renewable – 408 kJeq Scenario 3: Current – 588.2 kJeq Renewable – 456.9 kJeq	Per kilometer	Mopeds	Wortmann et al. (2021)
Ecosystem damage	Eutrophication	Scenario 1: Current 0.035/Renewable 0.0152 g Peq Scenario 2: Current 0.0356/Renewable 0.0152 g Peq Scenario 3: Current 0.0392/Renewable 0.0192 g Peq	Per kilometer	Mopeds	Wortmann et al. (2021)
	Ecosystem damage (Species years)	Shared bike: 1.25 × 10^−9^ species years Private bike: 4.65 × 10^−10^ species years Shared e-scooter: 2.29 × 10^−9^ species years Private e-scooter: 2.33 × 10^−9^–1.65 × 10^−9^ species years Shared moped: 1.64 × 10^−9^ species years Private motorcycle: 4.03 × 10^−9^ species years	Per kilometer	Micromobility sharing	de Bortoli (2021)

**Table 9.** List of factors influencing environmental impacts from shared mobility and their categorisation.

Factor	Category
Modal shift due to shared mobility	Travel behaviour
Changes in kilometers traveled by vehicle	
Changes in vehicle occupancy	
Rebalancing strategy of the shared fleet	Design and operation of transportation modes
Service life of vehicles in the shared fleet	
Number of shared vehicles in the system (utilisation rate)	
Technology used in the shared fleet	
Parking strategy (dockless and docked micromobility sharing systems/free-floating and stationary car sharing)	
Materials used during the manufacturing of the shared vehicle	
End-of-life of the shared vehicles	
Purchase of private vehicles	Consumption
Electricity mix used to charge vehicles	Context

### 3.1. Environmental impacts of passenger car sharing

#### 3.1.1. Climate impacts

Car sharing has the potential to both reduce and increase emissions from passenger transportation. Emissions from car sharing ranged between 79.6 and 283.2 g carbon dioxide equivalent (CO_2_eq) per passenger-kilometer (pkm), while private driving emissions ranged between 244.7 and 250 g CO_2_eq pkm (Table 4) (Chen & Kockelman, 2016; Sun & Ertz, 2021b). When emissions were analyzed at the annual per-person level, emissions decreased by 0.08–0.94 t CO_2_eq or increased by 0.02–0.25 t CO_2_eq (Table 4) (Amatuni et al., 2020; Arbeláez Vélez & Plepys, 2021; Firnkorn & Müller, 2011; Martin & Shaheen, 2011; Migliore et al., 2020; Namazu & Dowlatabadi, 2015).

Most of the emissions occurred during the use phase, followed by the production phase; emissions during EoL and infrastructure building were minor. Emissions during the use phase account for 40%–95% of total emissions and varied depending on how people changed their travel behaviour and the way in which the car sharing scheme was designed and implemented (Amatuni et al., 2020; Chen & Kockelman, 2016; Ding et al., 2019; Lausselet et al., 2021; Raugei et al., 2021; Sun & Ertz, 2021b).

Changes in travel behaviour due to car sharing are measured through variations in vehicle-kilometers traveled, modal split, vehicle occupancy, and vehicle ownership rates (Amatuni et al., 2020; Arbeláez Vélez & Plepys, 2021; Caulfield, 2009; Farrell et al., 2010; Firnkorn & Müller, 2011; Lausselet & Brattebø, 2021; Migliore et al., 2020; Sun & Ertz, 2021b). Lausselet and Brattebø (2021) reported that emissions in a specific neighborhood could be reduced from 41,675 to 34,486 t CO2eq per year by increasing the use of public transportation, reducing kilometers traveled, and increasing vehicle occupancy. In Palermo, car sharing reduced CO_2_ emissions from 334.5–208.9 t over a 10 month period due to increased use of public transportation and a reduction in vehicle ownership after car sharing became available in the city (Migliore et al., 2020). Nonetheless, there was an increase in methane (CH_4_) emissions – from 0.03 to 0.1 t – because the assessed shared fleet included vehicles fueled by diesel(Migliore et al., 2020). In the Netherlands, after car sharing became available, travelers increased their use of trains, buses, and bikes by 14.2%, 1.4% and 1.0%, respectively, decreasing transportation-related emissions by 823 kg CO2eq per person-year (Amatuni et al., 2020).

Emissions during the use phase are influenced by the design and operation of car sharing schemes – specifically parking and rebalancing strategies – as well as the vehicle technology used in the shared fleet; the form of ownership (B2C vs. P2P) was less relevant. Rebalancing of the fleet refers to the process of relocating the shared vehicles to specific locations, meaning that vehicles are driven without a passenger. In Ding et al. (2019), emissions from car sharing ranged from 2249 to 4549 kg CO_2_ per person-year, with the lower end corresponding to stationary schemes that use the same drop-off and pick-up station, and the higher emission range corresponding to stationary schemes that offered several stations. In addition to parking strategy, the wide variation in emissions also reflects the rebalancing of the fleet, the number of vehicles in the shared system, and vehicle occupancy.

The analysis identified the type of vehicles used in the shared fleet as another factor influencing climate impacts. Baptista et al. (2014) calculated the annual impact from car sharing in Lisbon to be 9.5 t of CO_2_ per year. This impact could be reduced by 35%–65% if the fleet shifted to hybrid or electric vehicles, respectively. When a shared fleet includes electric or hybrid vehicles, the energy source for electricity was identified as one key factor in determining climate impacts (Schelte et al., 2021; Zhang et al., 2021). Other studies suggest that shared vehicles are more fuel efficient than privately owned ones. Chen and Kockelman (2016) showed that the greater fuel efficiency of shared fleet vehicles and changes in travel behaviour could result in a reduction in emissions from 244.7 to between 80.0 and 163.7 g CO2eq per pkm.

Regarding the form of ownership of shared vehicles, Arbeláez Vélez and Plepys (2021) report that B2C and P2P car sharing produce similar emissions during the use phase. When users shift from public transit and active transportation to car sharing, emissions increase by 23.4–25.7 kg of CO_2_eq per person-year for B2C and P2P respectively. Meanwhile, people who forego private vehicle ownership to engage in car sharing decrease their emissions by 924.8 and 941.5 kg of CO_2_eq per person-year, for B2C and P2P car sharing, respectively.

Carpooling also led to decreased climate impacts due to an increase in vehicle occupancy. In Ireland, estimated savings related to vehicle occupancy ranged from 9.0 to 30.0 t CO_2_. This wide range reflects different scenarios based on individual and household characteristics (Table 5) (Farrell et al., 2010). Caulfield (2009) estimated the potential annual savings in Dublin could range from 7604 to 12,674 t CO_2_ per year, depending on the number of days that people were willing to carpool to work.

Emissions during the production phase account for 3%–56% of total life-cycle emissions (Amatuni et al., 2020; Chen & Kockelman, 2016; Ding et al., 2019; Lausselet et al., 2021; Raugei et al., 2021; Sun & Ertz, 2021b). Emissions in this phase are connected to the length of time that shared vehicles are in the shared system, their utilisation rate, and the type of vehicles used (Amatuni et al., 2020; Chen & Kockelman, 2016; Ding et al., 2019; Sun & Ertz, 2021b).

Although changes in expenditure due to passenger car sharing are not often considered in the reviewed literature, this is another factor that influences climate impacts. For example, if people foregoes their car they might change their expenditure in fuel, car maintenance and insurance. Ma et al. (2018) found that carpooling’s potential CO_2_ emissions reduction in Beijing was 612.8 × 10^3^ t due to lower vehicle ownership rates and modal shifts. Schelte et al. (2021) proposed that the quantification of environmental impacts should consider household or individual mobility budgets before and after they start using shared mobility.

#### 3.1.2. Resource depletion

Resource depletion was measured from two perspectives: fuel and material. Impacts from fuel depletion occurred mostly in the use phase and showed a decrease, largely linked to greater efficiency of vehicles in the shared fleets, modal shifts, and a reduction in distances traveled (Table 4 and Table 5) (Baptista et al., 2014; Chen & Kockelman, 2016; Lausselet & Brattebø, 2021; Ma et al., 2018; Te & Lianghua, 2020; Yu et al., 2017; Zhang et al., 2021). In Lisbon, energy use from car sharing could be reduced from 125 to 82 or 67 GJ per year if vehicles were hybrid or electric, respectively (Baptista et al., 2014). Studies focusing on carpooling in specific Chinese cities reported energy savings of 196 × 10^3 ^MJ over three months (Yu et al., 2017).

One study assessed material depletion, finding that the majority of impacts occurred during the manufacturing of vehicles in the shared fleet and accounted for 40%–55% of total impacts. Lausselet and Brattebø (2021) estimated savings of 6.1–43 t Fe-eq due to carpooling in a neighborhood over a one-year period as a result of increased vehicle occupancy and extended use of the vehicles in the sharing system fleet (Table 5).

Building new infrastructure was found to have a limited influence on fuel and material depletion, mostly because the infrastructure needed for shared-car use was already in place in the cases analyzed (Lausselet & Brattebø, 2021). Although it is relevant for the analysis of environmental impacts, impacts specifically during the shared car EoL stage have yet to be studied.

#### 3.1.3. Air pollution

The reviewed assessments only calculated air pollution emitted during the use phase, where it depends on the type of vehicle used in the shared fleet and the degree of modal shift among users. For example, in Palermo, an increase of 0.0122 t of NO_x_ per year and a decrease of 0.007 t of PM_10_ per year was calculated, because the car sharing fleet used diesel vehicles (see Table 4) (Migliore et al., 2020).

#### 3.1.4. Other impact categories

A decrease in vehicle ownership due to car sharing has the potential of reducing land use during the use phase by 4.68 × 10^9^ m^2^ per year in China (Table 4) (Te & Lianghua, 2020). Other impacts, such as ozone depletion, freshwater eutrophication, and terrestrial acidification, decrease due to changes in travel behaviour and the use of more efficient vehicles.

Carpooling can potentially generate savings in freshwater eutrophication and terrestrial acidification of 178.0–254.4 t-eq SO_2_ and 17.27–21.3 kg P-eq, respectively (Table 5) (Lausselet & Brattebø, 2021). Ozone depletion was reported to decrease (Migliore et al., 2020). These reductions are associated with increased car occupancy.

### 3.2. Micromobility sharing

#### 3.2.1. Climate impacts

Emissions from shared micromobility systems are higher than emissions from private micromobility usage. Emissions ranged from 57 to 68 g CO_2_eq per km for docked bikesharing, from 118 to 129 g CO_2_eq per km for dockless bikesharing, from 61 to 109 g CO_2_eq per km for e-scooter sharing, and from 20 to 119 g CO_2_eq per km for shared mopeds. Emissions from private biking, in turn, ranged from 7.47 to 11.7 g CO_2_eq per km (Table 6, Table 7, and Table 8) (Bonilla-Alicea et al., 2020; de Bortoli & Christoforou, 2020; Hollingsworth et al., 2019; Kazmaier et al., 2020; Luo et al., 2019; Schelte et al., 2021; Wortmann et al., 2021).

Most of the emissions from shared micromobility were caused in the production and use phases (specifically fleet rebalancing and maintenance). The vehicle production phase accounted for ranged 28–90% of emission, while the use phase ranged accounted for 10%–70% (Bonilla-Alicea et al., 2020; de Bortoli & Christoforou, 2020; Hollingsworth et al., 2019; Kazmaier et al., 2020; Luo et al., 2019; Schelte et al., 2021; Wortmann et al., 2021). Emissions from the production of docking stations accounted for 23% of total emissions, while emissions from EoL processes were minor (Bonilla-Alicea et al., 2020; de Bortoli & Christoforou, 2020; Hollingsworth et al., 2019; Kazmaier et al., 2020; Luo et al., 2019; Schelte et al., 2021; Wortmann et al., 2021).

Emissions during the production phase were linked to factors such as the number of shared vehicles and the length of their service in the shared system, as well as their utilisation rates (Bonilla-Alicea et al., 2020; Hollingsworth et al., 2019; Kazmaier et al., 2020; Moreau et al., 2020). Tao and Zhou (2021) evaluated the impacts of free-floating bikesharing and found that GWP could be reduced by 23.9 g CO_2_eq pkm if bikes achieve a high utilisation rate, compared with a reduction of 13.4 g CO_2_eq pkm when there is low utilisation. Kazmaier et al. (2020) reported that emissions from shared e-scooters could be reduced from 165 to 97 g CO_2_eq pkm if the service life for each scooter in the shared system increased from 2117 km (equivalent to six months of service) to 4057 km (equivalent to 15 months). Moreau et al. (2020) also found that emissions would be reduced by extending the service life of vehicles.

During the use phase, most of the emissions came from the rebalancing and maintenance strategy (Bonilla-Alicea et al., 2020; Luo et al., 2019; Sun & Ertz, 2021b; Tao & Zhou, 2021; Wang et al., 2021). Hollingsworth et al. (2019) argue that sharing organisations can reduce climate impacts by using fuel-efficient vehicles for rebalancing, optimising routes (and thereby reducing driving distances), and only collecting vehicles that need to be recharged. In their estimations, rebalancing accounts for 43% of CO_2_ emissions from micromobility sharing, while materials and manufacturing account for 50%. A reduction of approximately 1 km in the distance traveled to pick-up e-scooters would result in a 27% reduction in emissions, while using fuel efficient vehicles for rebalancing would result in a 12% reduction. Luo et al. (2020) present similar outcomes for efficient rebalancing strategies and highlight the need for more depots for charging and servicing shared vehicles. de Bortoli (2021) found that if electric vans were used for rebalancing in countries with a low-carbon energy mix, the distance driven would lose its significance. In her results, de Bortoli (2021) showed that the length of trips needed to rebalance the shared system is more relevant for e-scooters, given that they require more servicing (rebalancing, maintenance, and charging) than bikes or mopeds. Although the results of these studies show some disparities regarding which variable should be prioritised in an efficient rebalancing strategy, they largely agree that distance driven, the characteristics of vehicles used for rebalancing, and the energy used for charging are the most important factors.

During the use phase, special attention needs to be given to the type of transportation that shared micromobility replaces. When e-scooter, bike, and moped sharing replaces trips made by more emission-intensive transportation alternatives, such as private driving, it has the potential to reduce emissions. However, if micromobility replaces active transportation modes, emissions are more likely to increase (Ding et al., 2021; Hollingsworth et al., 2019).

#### 3.2.2. Resource depletion

Resource depletion was assessed in terms of depletion of fuel and materials. For fuel depletion, impacts occurring during the production phase account for 60%–78% of total impacts, while those in the use phase account for 40%–70%. The EoL phase has limited impacts (de Bortoli, 2021; Moreau et al., 2020; Wang et al., 2021; Wortmann et al., 2021). Fuel depletion was driven by the need for additional infrastructure (such as the share bike docks) and influenced by the service life and utilisation rate of the vehicles in the shared system, as well as by the rebalancing strategy. In the case of stationary shared bikes, 68% of fuel depletion comes from the manufacturing of stations. In the case of e-scooters, manufacturing the vehicle and the infrastructure it needs account for 51% and 26% of fuel depletion impacts, while 21% of these impacts come during use of the vehicle (new cycle lanes for e-scooters). In the case of mopeds, 28% of fuel depletion comes from vehicle production, 48% from its use stage and 25% from the need to build new infrastructure (de Bortoli, 2021).

In the case of material depletion, the parking strategy was crucial in determining impacts. Stationary bikes consume more steel and aluminium than free-floating bikes due to the materials used to produce the stations. Stationary bikes consumed between 5.9 g to almost 12 g of Al per trip compared to free-floating shared bikes, which consume between 3.8 and 7 g of Al per trip. In the case of steel, stationary bikes consume between approximately 15 and 30 g of steel per trip compared to free-floating shared bikes, which consume between 2.5 and 6 g of steel per trip (Sun & Ertz, 2021a). No study has yet compared material depletion for shared bikes versus privately owned bikes.

Resource depletion varies depending on the service life of shared vehicles in the system and their utilisation rate. Moreau et al. (2020) estimated that copper depletion could decrease from 2 g pkm to 0.6 to 1.3 g pkm if the service life of e-scooters were extended from 1 year to 2.5 years. Tao and Zhou (2021) estimated that the abiotic resource depletion of minerals, fossil fuels, and renewable energies would decrease if the utilisation rate of shared bikes rose from 3285 to 13,140 km during its lifetime.

#### 3.2.3. Air pollution

The shared vehicle production phase accounts for 67%–78% of air pollution, followed by the use phase. EoL accounted for only a limited amount of air pollution. Factors directly connected to air pollution include the size of the fleet, the source of the energy used to charge the vehicles, the service life time of the vehicles, and the rebalancing strategy (Hollingsworth et al., 2019; Moreau et al., 2020; Wortmann et al., 2021). Extending the service life of e-scooters in the shared system by 2.5 years could decrease PM_2.5_ emissions from 0.3 to 0.1 g of PM_2.5_ pkm (Moreau et al., 2020). If mopeds are charged using renewable energy, air pollution can be reduced from 0.06–0.05 g PM_2.5_ pkm (Wortmann et al., 2021).

#### 3.2.4. Other impact categories

The production of the shared vehicles accounts for 53%–73% of acidification and eutrophication (Hollingsworth et al., 2019). Factors such as the service life of vehicles in the shared system and frequency of battery replacement also influenced these impacts (Hollingsworth et al., 2019; Wortmann et al., 2021).

Luo et al. (2019) introduced an aggregate indicator for ozone depletion, acidification, eutrophication, smog formation, ecotoxicity, and resource use to evaluate docked and dockless bikesharing schemes compared to other transportation modes. They found that docked and dockless bikes have higher impacts than privately owned bikes. For stationary bikesharing, 61% of impacts came from the docks themselves, while for dockless bikesharing the manufacturing of the bicycle and rebalancing were responsible for 52% and 39% of impacts, respectively.

### 3.3. Summary of factors influencing the environmental impacts of shared mobility

Table 9 summarises the factors that influence the environmental impacts from shared mobility modes, grouped into the four categories of factors that emerged in this review.

## 4. Discussion

The results of this review show that shared mobility can be part of either the solution or the problem as countries seek to decrease environmental impacts from passenger transportation. The results show that the idea that negative environmental impacts from passenger transportation will unconditionally decrease as the expansion of shared mobility modes enables a shift away from private car ownership to vehicle sharing is misleading. Instead it was found that the environmental outcomes are more complex and that factors such as the design and operation of shared modes and the specificities of the context, and travel behaviour influence the environmental impacts of shared mobility.

Local governments, sharing organisations, and users all have roles in shaping the environmental impacts of shared mobility. Local governments that allow new transportation modes to be offered should define the role of new transportation modes in the transportation system, the goals that new transportation modes will help achieve, and how the local government will engage in the process. Reflecting on these questions can help cities clarify whether a new transportation mode is meant to cover the first and last miles, for example, or whether a new mode is meant to be used when people need to travel to isolated areas. Local governments can also define the role that they will play when interacting with sharing organisations, such as regulating, collaborating, or providing financial support or subsidies for them (Voytenko Palgan et al., 2021).

The tension between profitability and reducing environmental impact is a challenge in designing and implementing shared mobility systems that have the best chance of decreasing environmental impacts from passenger transportation (Santos, 2018). One example of this is when shared mobility systems use vehicles with low utilisation rates; this leads to an oversupply of vehicles, which exacerbates the negative impacts from vehicle manufacturing. Local governments that regulate, collaborate, enable, and support sharing organisations can be more proactive in managing this tension and ensuring that the shared mobility systems deployed in their cities genuinely help decrease environmental impacts from passenger transportation (Voytenko Palgan et al., 2021).

Shared mobility may cause trade-offs between different environmental impacts or between environmental and social impacts. To understand these trade-offs and mitigate their negative consequences, shared mobility organisations should be encouraged to share data about their operations and impacts with local governments. This would enable the local governments to make informed decisions regarding the transportation needs of their citizens and form a better idea of what is or is not likely to work in their context.

Each specific context will differ in terms of how shared mobility solutions will integrate into the existing transportation system and which transportation modes it replaces or complements. Replicating shared mobility solutions from other locations without considering contextual variables is unlikely to yield identical results in the new context. Local governments that implement shared mobility solutions with a clear objective can monitor the implementation and operation of shared mobility solutions and evaluate possible adjustments so shared mobility enables them to achieve these goals.

Context variables such as the built environment, cultural beliefs, and income levels have been shown to influence how people choose to travel (Ding et al., 2017; Ding et al., 2018). For example, cities that have a biking tradition and culture have a higher biking modal share than cities where there is no biking culture. Cities that are densely populated might have higher usage of public transportation networks. Thus, it is likely that citizens of cities where there is a biking tradition and culture could adopt micro-mobility and use this transport service as first and last mile. However, research that explores how contextual variables influence the environmental impacts of shared mobility needs to be synthesised to give solid evidence that local governments and shared organisations can use to develop environmentally sound shared mobility services.

### 4.1. Hotspots of environmental impact during the lifecycle of a shared mobility system

In our analysis we identify specific life-cycle phases (and activities within these life-cycle phases) that are “hotspots” in terms of environmental impacts for each shared mobility service. In this section we discuss these phases and activities, as well as strategies to mitigate their impacts.

#### 4.1.1. Passenger car sharing

The use phase was identified as a hotspot for climate impacts, air pollution, and fuel depletion. This result is in line with other studies that explore the impacts of car ownership and use (Helmers & Marx, 2012; Ivanova et al., 2020; Messagie et al., 2014). A switch from internal combustion engine (ICE) to electric vehicles can mitigate greenhouse gas emissions and fuel depletion, as well as improve local air quality (Helmers & Marx, 2012; Ivanova et al., 2020; Wynes & Nicholas, 2017). These benefits are achieved if electric cars are charged using renewable energy (Ellingsen et al., 2016). Therefore, cities should prioritise the implementation of car sharing systems that use electric shared fleets that can be charged with renewable energy (Lausselet et al., 2021; Raugei et al., 2021; Te & Lianghua, 2020).

There are other factors in the design and operation of shared mobility systems that affect climate, fuel, and air quality environmental impacts in the use phase, namely rebalancing and parking strategies. Shared systems that use ICE cars that must be driven long distances to rebalance fleet distribution performed poorly in terms of environmental impacts (Luo et al., 2020). Although these impacts are lower for electric shared vehicle fleets, urban sharing organisations and local governments should consider how the rebalancing and parking strategy can exacerbate other impacts, such as congestion.

Travel behaviour also influences impacts during the use phase. Car sharing and carpooling should be used as instruments to decrease car ownership and use. Some studies found that when people gained access to cars through car sharing, GHG emissions and fuel consumption could increase. However, these same people might never own their own cars, entailing a reduction in environmental impacts in other life-cycle phases such as production and EoL (Shaheen et al., 2019).

The number of shared cars in the system affects material depletion during the production phase. Shared cars should be available in the adequate numbers and in the right locations to meet demand without an oversupply. Shared systems with an oversupply of vehicles can be identified through the low utilisation rates of the fleet’s cars. Cities that have access to information about utilisation rates should establish minimum service lives for cars in the shared car fleet.

Our analysis did not identify significant environmental impacts from other life-cycle phases of shared cars, such as EoL or building infrastructure. Automobile infrastructure has considerable environmental impacts, and if car sharing or carpooling increase in scale, the impacts of such car infrastructure could become more relevant and would need to be mitigated.

#### 4.1.2. Micromobility sharing

This analysis found that the production of vehicles and docking stations for micromobility sharing systems was the life-cycle phase that contributed the most to climate impacts, air pollution, acidification, eutrophication, fuel, and material depletion. The short service lives of shared vehicles, as well as a tendency towards oversupply, were major contributors that aggravated these environmental impacts. These variables can be controlled by the sharing organisation, but this runs up against the previously mentioned tension between profit and environmental impacts (Santos, 2018). The livability of cities has been affected by the oversupply of micromobility share vehicles, and as a result some local governments have put caps on the number of vehicles that sharing organisations can make available (City of Melbourne, 2022). Although this measure is effective in mitigating impacts related with livability, it does not address environmental issues in a systematic way. Sharing organisations should improve the durability of their vehicles, and local governments should establish minimum requirements for the durability and service life of shared vehicles.

Rebalancing and maintenance were two activities that influenced the impacts caused during the use phase, with particular influence on climate impacts and fuel depletion. Shared fleets should be rebalanced using electric vehicles and in this way the distance driven to rebalance the fleet would no longer be critical in increasing environmental impacts. In the case of battery-powered modes, how often batteries are replaced was another variable that affected system impacts. This means that sharing organisations should work toward the development of shared vehicles and batteries that are more durable.

The behaviours of shared micromobility vehicle users influences how often vehicles need to be maintained or replaced. Several cities report that Shared fleet vehicles are often vandalised or misused (for example, carrying a second person on an e-scooter). Users must be educated about how to correctly use such vehicles in order to minimise environmental impacts.

#### 4.1.3. Future research

Research gaps with respect to environmental impacts are changes in land use, material depletion, or harm to ecosystems due to the use of shared mobility. In the case of material depletion, differences between B2C and P2P car sharing schemes have not been studied, and the differences in climate impacts between these two ownership models has only received a limited amount of attention that focused during the use phase (Arbeláez Vélez & Plepys, 2021).

Since shared mobility has the potential to change household incomes and transportation expenditures, we need more research on possible rebound effects. Studies might explore how other characteristics of business models, such as payment or membership schemes, might affect environmental impacts in a way that further contributes to building sharing organisations more likely to decrease impacts from passenger transportation.

## 5. Conclusion

This literature review has aimed to give an overview of the environmental impacts of car sharing, carpooling, bikesharing, and scooter/moped sharing and has also sought to explore the factors that influence these impacts. Broadly, this study contributes to the field by providing a structured synthesis of current knowledge that can help build shared mobility systems that decrease environmental impacts from passenger transportation. It also contributes by providing guidelines for local governments and shared mobility organisations to use when designing and implementing shared mobility systems in their cities. More specifically, this study contributes by (1) expanding and updating existing understandings of the environmental impacts of shared mobility and (2) identifying and grouping the factors that influence the environmental impacts of shared mobility. These contributions also support the development of transportation policies that ultimately must focus on incentivising the use of active transportation (suck as cycling and walking) and public transportation while discouraging private car ownership.

Shared mobility holds the potential to either exacerbate or reduce environmental impacts from passenger transportation. Its effect on environmental impacts is influenced by factors that determine whether its implementation will produce gains or losses, including travel behaviour, the design and operation of shared mobility systems, and contextual characteristics. We need studies that contribute to a more holistic and systemic understanding of the environmental impacts of shared mobility in order to develop more robust urban transportation systems.

## Supplementary Material

Supplemental Material

Supplemental Material

Supplemental Material

Supplemental Material
