# Supplementary material for: Environmental impacts of shared mobility: a systematic literature review of life-cycle assessments focusing on car sharing, carpooling, bikesharing, scooters and moped sharing
Source: Transp Rev. 2023 Nov 13;44(3):634–58. doi: 10.1080/01441647.2023.2259104 (PMC10962713; doi:10.1080/01441647.2023.2259104)
Supplement: Supplemental Material [file TTRV_A_2259104_SM7893.pdf]

Ref	Title	Author	Year	Journal	Level of analysis	Geographical location	Sub type of shared mobility
1	Carbon Emission Effect of the Dock-Less Bike-Sharing System in Beijing from the Perspective of Life Cycle Assessment	Ding, Qingjiang; Li, Jinghui; Wang, Qixuan; Li, Chunhui; Yue, Wencong	2021	Journal of Environmental Accounting and Management	City	Asia	Dockless shared bikes
2	LCA and scenario analysis of a Norwegian net-zero GHG emission neighbourhood: The importance of mobility and surplus energy from PV technologies	Lausselet, C.; Lund, K. M.; Brattebo, H.	2021	Building and Environment	Neighborh	Other	Car-sharing
3	Contribution of bike-sharing to urban resource conservation: The case of free-floating bike-sharing	Sun, Shouheng; Ertz, Myriam	2021	Journal of Cleaner Production	City	Asia	Dockless shared bikes
4	Life-Cycle Assessment of Carbon Footprint of Bike-Share and Bus Systems in Campus Transit	Wang, Sishen; Wang, Hao; Xie, Pengyu; Chen, Xiaodan	2021	Sustainability (Switzerland)	Region	North Ame	NA
5	Techno-Economical and Ecological Potential of Electrical Scooters: A Life Cycle Analysis	Kazmaier, Markus; Taefi, Tessa; Hettesheimer, Tim	2020	European Journal of Transport and Infrastructure Research	City	Europé	NA
6	Consequential LCA for territorial and multimodal transportation policies: method and application to the free-floating e-scooter disruption in Paris	de Bortoli, Anne; Christoforou, Zoi	2020	Journal of Cleaner Production	City	Europé	NA
7	Life cycle carbon dioxide emissions of bike sharing in China: Production, operation, and recycling	Chen, Jingrui; Zhou, Dan; Zhao, Yue; Wu, Bohong; Wu, Tian	2020	Resources, Conservation and Recycling	Country	Asia	Dockless
8	Does car sharing reduce greenhouse gas emissions? Assessing the modal shift and lifetime shift rebound effects from a life cycle perspective	Amatuni, Levon; Ottelin, Juudit; Steubing, Bernhard; Mogollon, Jose M.	2020	Journal of Cleaner Production	Individual	USAEurope	B2C
9	Optimizing bike sharing systems from the life cycle greenhouse gas emissions perspective	Luo, Hao; Zhao, Fu; Chen, Wei-Qiang; Cai, Hua	2020	Transportation Research Part C: Emerging Technologies	City	Asia	Dockless
10	Dockless E-Scooter: A Green Solution for Mobility? Comparative Case Study between Dockless E-Scooters, Displaced Transport, and Personal E-Scooters	Moreau, Helie; de Meux, Loic de Jamblinne; Zeller, Vanessa; D'Ans, Pierre; Ruwet, Coline; Achten, Wouter M. J.	2020	Sustainability (Switzerland)	City	Europé	NA
11	Life cycle assessment to quantify the impact of technology improvements in bike-sharing systems	Bonilla-Alicea, Ricardo Javier; Watson, Bryan C.; Shen, Ziheng; Tamayo, Laura; Telenko, Cassandra	2020	Journal of Industrial Ecology	City	Other	Dockles and docked shared bikes
12	Carsharing: mitigation strategy for transport-related carbon footprint	Qi Te; Chen Lianghua	2020	Mitigation and Adaptation Strategies for Global Change	Country	Asia	Not specify
13	Life cycle assessment of car sharing models and the effect on GWP of urban transportation: A case study of Beijing	Ding, Ning; Pan, Jingjin; Zhang, Zhan; Yang, Jianxin	2019	Science of the Total Environment	City	Asia	Fre- flotingStationary based
14	Sustainable consumption in mobility from a life cycle assessment perspective	Severis, Roni M.; Simioni, Flavio J.; Moreira, Jose Mauro M. A. P.; Alvarenga, Rodrigo A. F.	2019	Journal of Cleaner Production	Individual	Latin Amer	CarpoolingCar-sgaring
15	Are e-scooters polluters? The environmental impacts of shared dockless electric scooters	Hollingsworth, Joseph; Copeland, Brenna; Johnson, Jeremiah X.	2019	Environmental Research Letters	Business m	North Ame	NA
16	Comparative life cycle assessment of station-based and dock-less bike sharing systems	Luo, Hao; Kou, Zhaoyu; Zhao, Fu; Cai, Hua	2019	Resources, Conservation and Recycling	City	North Ame	Dockless and docked

17	Spatial Heterogeneous Characteristics of Ridesharing in Beijing-Tianjin-Hebei Region of China	Ma, Ye; Yu, Biying; Xue, Meimei	2018	Energies	City	Asia	NA
18	Environmental benefits of bike sharing: A big data-based analysis	Zhang, Yongping; Mi, Zhifu	2018	Applied Energy	City	Asia	Dockless
19	Environmental benefits from ridesharing: A case of Beijing	Yu, Biying; Ma, Ye; Xue, Meimei; Tang, Baojun; Wang, Bin; Yan, Jinyue; Wei, Yi-Ming	2017	Applied Energy	City	Asia	NA
20	Carsharing's life-cycle impacts on energy use and greenhouse gas emissions	Chen, T. Donna; Kockelman, Kara M.	2016	Transportation Research Part D: Transport and Environment	Country	North Ame	NA
21	Characterizing the GHG emission impacts of carsharing: a case of Vancouver	Namazu, Michiko; Dowlatabadi, Hadi	2015	Environmental Research Letters	City	North Ame	NA
22	Greenhouse Gas Emission Impacts of Carsharing in North America	Martin, Elliot W.; Shaheen, Susan A.	2011	IEEE Transactions on Intelligent Transportation Systems	Household	North Ame	NA
23	What will be the environmental effects of new free-floating car-sharing systems? The case of car2go in Ulm	Firnkorn, Joerg; Mueller, Martin	2011	Ecological Economics	City	Europé	NA
24	Estimating the Potential Success of Sustainable Transport Measures for a Small Town	Farrell, Seona; McNamara, David; Caulfield, Brian	2010	Transportation Research Record	City	Europé	NA
25	Evaluation of Potential Contribution of Dockless Bike-sharing Service to Sustainable and Efficient Urban Mobility in China	Tao J., Zhou Z.	2021	Sustainable Production and Consumption	City	Asia	Dockless
26	Environmental impact of mutualized mobility: Evidence from a life cycle perspective	Sun S., Ertz M.	2021	Science of the Total Environment	City	Asia - Nort	Dockless and docked, Ridesharing, carpooling and car-shairng
27	High-resolution assessment of environmental benefits of dockless bike-sharing systems based on transaction data	Li A., Gao K., Zhao P., Qu X., Axhausen K.W.	2021	Journal of Cleaner Production	City	Asia	Dockless bikes
28	Study on Life-Cycle Energy Impact of New Energy Vehicle Car-Sharing with Large-Scale Application	Zhang B., Lu Q., Wu P.	2021	Journal of Energy Storage	City	Asia	NA
29	Environmental performance of shared micromobility and personal alternatives using integrated modal LCA	de Bortoli A.	2021	Transportation Research Part D: Transport and Environment	Business m	Europé	NA
30	Car Sharing as a Strategy to Address GHG Emissions in the Transport System: Evaluation of Effects of Car Sharing in Amsterdam	Arbelaez Velez, A.M., Plepys, A,.	2021	Sustainability (Switzerland)	City/Indivic	Europé	p2p and B2C
31	The environmental benefits of carsharing: the case study of Palermo.	Migliore M, D'Orso G, Caminiti D	2020	Transportation Research Procedia	City	Europé	Stationary base
32	Energy, Environmental and Mobility Impacts of Car-sharing Systems	Baptista, P.; Melo, S.; Rolim, C.	2014	Procedia - Social and Behavioral Sciences	City	Europé	Stationary base
33	Assessing the Carbon Impact of ICT Measures: A Case Study Investigation Using Latis1 Model	Stewart K.	2015	International Journal of Transportation Science and Technology	Region	Europé	NA
34	The IMOSMID project: Increasing energy efficiency by identification, assessment and use of eco-friendly technologies and management plans for public and private transport	Buzzoni L.	2013	The Sustainable City VIII	Region	Europé	NA

35	Estimating the environmental benefits of ride-sharing: A case study of Dublin	Caulfield B.	2009	Transportation Research Part D	Country	Europé	NA
36	Emissions assessment of bike sharing schemes: The case of Just Eat Cycles in Edinbur	D'Almeida L., Rye T., Po	2021	Sustainabl	City	Europé	Stationary base
37	Analysis of electric moped scooter sharing in berlin: A technical, economic and enviro	Wortmann C., Syré A.M	2021	World Elec	City	Europé	Dockless
38	Environmental implications of the ongoing electrification of the UK light duty vehicle	Raugei M., Kamran M.,	2021	Resources,	Country	Europé	Car-sharing/Ridesharing
39	Life cycle assessment on electric moped scooter sharing	Schelte N., Severengiz S	2021	Sustainabil	Business m	Europé	NA
40	Environmental co-benefits and trade-offs of climate mitigation strategies applied to net-zero-emission neighbourhoods	Lausselet C., Brattebø H.	2021		Neighborh	Europé	NA
