# Supplementary material for: Environmental impacts of shared mobility: a systematic literature review of life-cycle assessments focusing on car sharing, carpooling, bikesharing, scooters and moped sharing
Source: Transp Rev. 2023 Nov 13;44(3):634–58. doi: 10.1080/01441647.2023.2259104 (PMC10962713; doi:10.1080/01441647.2023.2259104)
Supplement: Supplemental Material [file TTRV_A_2259104_SM7850.pdf]

Annex 1: Table with the coded information about the papers as well as the extraction of the results

Annex 2: Information about the environmental impacts measured in each of the papers with the classification

Inventory: In Annex 1 each papers has a number under the column reference. This worksheets has all the information about the paper
