# Supplementary material for: Environmental impacts of shared mobility: a systematic literature review of life-cycle assessments focusing on car sharing, carpooling, bikesharing, scooters and moped sharing
Source: Transp Rev. 2023 Nov 13;44(3):634–58. doi: 10.1080/01441647.2023.2259104 (PMC10962713; doi:10.1080/01441647.2023.2259104)
Supplement: Supplemental Material [file TTRV_A_2259104_SM7799.pdf]

Title	Environmental impact	Standard environmental impact
Carbon Emission Effect of the Dock-Less Bike-Sharing System in Beijing from the Perspective of Life Cycle Assessment	CO2 emissions	Climate change
LCA and scenario analysis of a Norwegian net-zero GHG emission neighborhood: The importance of mobility and surplus energy from PV t	CO2 emissions	Climate change
Contribution of bike-sharing to urban resource conservation: The case of free-floating bike-sharing	Material depletion	Resource depletion
Life-Cycle Assessment of Carbon Footprint of Bike-Share and Bus Systems in Campus Transit	CO2 emissions	Climate change
Life-Cycle Assessment of Carbon Footprint of Bike-Share and Bus Systems in Campus Transit	Energy consumption	Resource depletion
Techno-Economical and Ecological Potential of Electrical Scooters: A Life Cycle Analysis	CO2 emissions	Climate change
Consequential LCA for territorial and multimodal transportation policies: method and application to the free-floating e-scooter disruption	C02 emissions	Climate change
Life cycle carbon dioxide emissions of bike sharing in China: Production, operation, and recycling	CO2 emissions	Climate change
Does car sharing reduce greenhouse gas emissions? Assessing the modal shift and lifetime shift rebound effects from a life cycle perspectiv	GHG emissions	Climate change
Optimizing bike sharing systems from the life cycle greenhouse gas emissions perspective	GHG emissions	Climate change
Dockless E-Scooter: A Green Solution for Mobility? Comparative Case Study between Dockless E-Scooters, Displaced Transport, and Persor	GWP	Climate change
Dockless E-Scooter: A Green Solution for Mobility? Comparative Case Study between Dockless E-Scooters, Displaced Transport, and Persor	Fine particulate matter	Air pollution
Dockless E-Scooter: A Green Solution for Mobility? Comparative Case Study between Dockless E-Scooters, Displaced Transport, and Persor	Mineral resource scarcity	Resource depletion
Dockless E-Scooter: A Green Solution for Mobility? Comparative Case Study between Dockless E-Scooters, Displaced Transport, and Persor	Fossil resource scarcity	Resource depletion
Life cycle assessment to quantify the impact of technology improvements in bike-sharing systems	GHG emissions	Climate change
Car sharing: mitigation strategy for transport-related carbon footprint	CO2 emissions	Climate change
Car sharing: mitigation strategy for transport-related carbon footprint	Energy consumption	Resource depletion
Car sharing: mitigation strategy for transport-related carbon footprint	Land use (parking area)	Land use
Life cycle assessment of car sharing models and the effect on GWP of urban transportation: A case study of Beijing	GWP	Climate change
Sustainable consumption in mobility from a life cycle assessment perspective	Eco points	Indicator
Are e-scooters polluters? The environmental impacts of shared dockless electric scooters	CO2 emissions	Climate change
Are e-scooters polluters? The environmental impacts of shared dockless electric scooters	PM2.5 emissions	Air pollution
Are e-scooters polluters? The environmental impacts of shared dockless electric scooters	SO2 emissions	Air pollution
Are e-scooters polluters? The environmental impacts of shared dockless electric scooters	N emissions	Air pollution
Comparative life cycle assessment of station-based and dock-less bike sharing systems	GHG	Climate change
Comparative life cycle assessment of station-based and dock-less bike sharing systems	Total normalized environment	Indicator
Spatial Heterogeneous Characteristics of Ridesharing in Beijing-Tianjin-Hebei Region of China	Fuel use	Resource depletion
Spatial Heterogeneous Characteristics of Ridesharing in Beijing-Tianjin-Hebei Region of China	CO2 emissions	Climate change
Spatial Heterogeneous Characteristics of Ridesharing in Beijing-Tianjin-Hebei Region of China	SO2 emissions	Air pollution
Spatial Heterogeneous Characteristics of Ridesharing in Beijing-Tianjin-Hebei Region of China	NOx emissions	Air pollution
Environmental benefits of bike sharing: A big data-based analysis	Fuel use	Resource depletion
Environmental benefits of bike sharing: A big data-based analysis	CO2 emissions	Climate change
Environmental benefits of bike sharing: A big data-based analysis	NOx emissions	Air pollution
Environmental benefits from ridesharing: A case of Beijing	Energy use	Resource depletion
Car sharing's life-cycle impacts on energy use and greenhouse gas emissions	Energy use	Resource depletion
Car sharing's life-cycle impacts on energy use and greenhouse gas emissions	GHG emissions	Climate change
Characterizing the GHG emission impacts of car sharing: a case of Vancouver	CO2 emissions	Climate change
Greenhouse Gas Emission Impacts of Car sharing in North America	GHG emissions	Climate change
What will be the environmental effects of new free-floating car-sharing systems? The case of car2go in Ulm	CO2 emissions	Climate change
Estimating the Potential Success of Sustainable Transport Measures for a Small Town	CO2 emissions	Climate change
Evaluation of Potential Contribution of Dockless Bike-sharing Service to Sustainable and Efficient Urban Mobility in China	ADP Water	Resource depletion
Evaluation of Potential Contribution of Dockless Bike-sharing Service to Sustainable and Efficient Urban Mobility in China	ADP minerals and fossil	Resource depletion
Evaluation of Potential Contribution of Dockless Bike-sharing Service to Sustainable and Efficient Urban Mobility in China	GWP	Climate change
Environmental impact of mutualized mobility: Evidence from a life cycle perspective	GWP	Climate change
High-resolution assessment of environmental benefits of dockless bike-sharing systems based on transaction data	GHG emissions	Climate change
Study on Life-Cycle Energy Impact of New Energy Vehicle Car-Sharing with Large-Scale Application	Energy Used	Resource depletion
Environmental impact of mutualized mobility: Evidence from a life cycle perspective	GWP	Climate change
Environmental performance of shared micro mobility and personal alternatives using integrated modal LCA	Climate change	Climate change
Environmental performance of shared micro mobility and personal alternatives using integrated modal LCA	Primary energy	Resource depletion
Environmental performance of shared micro mobility and personal alternatives using integrated modal LCA	Ecosystem damage	Ecosystem damage
Car Sharing as a Strategy to Address GHG Emissions in the Transport System: Evaluation of Effects of Car Sharing in Amsterdam	GHG emissions	Climate change
The environmental benefits of car sharing: the case study of Palermo.	CH4	Air pollution
The environmental benefits of car sharing: the case study of Palermo.	CO	Climate change
The environmental benefits of car sharing: the case study of Palermo.	CO2 emissions	Air pollution
The environmental benefits of car sharing: the case study of Palermo.	NOx emissions	Air pollution
The environmental benefits of car sharing: the case study of Palermo.	Ozone depletion	Air pollution
The environmental benefits of car sharing: the case study of Palermo.	PM10	Air pollution
Energy, Environmental and Mobility Impacts of Car-sharing Systems	Energy consumption	Resource depletion
Energy, Environmental and Mobility Impacts of Car-sharing Systems	CO2 emissions	Climate change
Assessing the Carbon Impact of ICT Measures: A Case Study Investigation Using Latis1 Model	CO2 emissions	Climate change
The IMOSMID project: Increasing energy efficiency by identification, assessment and use of eco-friendly technologies and management pl	CO2 emissions	Climate change
Estimating the environmental benefits of ride-sharing: A case study of Dublin	CO2 emissions	Climate change
Emissions assessment of bike sharing schemes: The case of Just Eat Cycles in Edinburgh, UK	CO2 emissions	Climate change
Analysis of electric moped scooter sharing in berlin: A technical, economic and environmental perspective	GWP	Climate change
Analysis of electric moped scooter sharing in berlin: A technical, economic and environmental perspective	Eutrophication potential	Ecosystem damage
Analysis of electric moped scooter sharing in berlin: A technical, economic and environmental perspective	Acidification potential	Ecosystem damage
Analysis of electric moped scooter sharing in berlin: A technical, economic and environmental perspective	Particulate Matter Formation	Air pollution
Environmental implications of the ongoing electrification of the UK light duty vehicle fleet	global warming potential (GWP)	Climate change
Environmental implications of the ongoing electrification of the UK light duty vehicle fleet	photochemical ozone creation potential (POCP),	Ozone creation
Environmental implications of the ongoing electrification of the UK light duty vehicle fleet	abiotic depletion potential (ADP),	Resource depletion
Life cycle assessment on electric moped scooter sharing	GWP	Climate change
Environmental co-benefits and trade-offs of climate mitigation strategies applied to net-zero-emission neighborhoods	GWP	Climate change
Environmental co-benefits and trade-offs of climate mitigation strategies applied to net-zero-emission neighborhoods	Freshwater Eutrophication	Ecosystem damage
Environmental co-benefits and trade-offs of climate mitigation strategies applied to net-zero-emission neighborhoods	Material Depletion	Resource depletion
Environmental co-benefits and trade-offs of climate mitigation strategies applied to net-zero-emission neighborhoods	Terrestrial acidification	Ecosystem damage
Environmental benefits from ridesharing: A case of Beijing	CO2 emissions	Climate change
Environmental benefits from ridesharing: A case of Beijing	NOx emissions	Air pollution
Environmental benefits from ridesharing: A case of Beijing	SO2 emissions	Air pollution
