# Supplementary material for: Environmental impacts of shared mobility: a systematic literature review of life-cycle assessments focusing on car sharing, carpooling, bikesharing, scooters and moped sharing
Source: Transp Rev. 2023 Nov 13;44(3):634–58. doi: 10.1080/01441647.2023.2259104 (PMC10962713; doi:10.1080/01441647.2023.2259104)
Supplement: Supplemental Material [file TTRV_A_2259104_SM7760.pdf]

| Reference | Shared mobility | Sub type of shared mobility | Environmental impact | Standard environmental impact | Level of analysis | Geographical location | System boundaries | Assessment | Comments about assessment | Comments about results | doi |
|---|---|---|---|---|---|---|---|---|---|---|---|
| 1 | Bike sharing | Dockless shared bikes | $CO_2$ emissions | Climate impacts | City | Asia | Production (excluding raw material extraction)<br>Use<br>Maintenance<br>Rebalancing | Assessment of the changes in emissions due to bike sharing in Beijing in one year | Different types of bikes are evaluates. They evaluate scenarios to explore effects of BSS substitution with other transport modes. Assume a substitution of travel by private car of 10%. | The modal shift that the user experiences is highly influential in the environmental impact. If the user shifts from private to bike the environmental effect is positive but if it changes from public transport to bike sharing it isn't. Rebalancing decrease the sustainability gains from bike sharing. Emissions from rebalancing are prominent. They mention that shared bikes aren't used enough. | 10.5890/JEAM.2021.03.004 |
| 2 | Car-sharing | Car-sharing | $CO_2$ emissions | Climate impacts | Neighborhood | Other | Use | Assessment of changes in emissions due to car-sharing in a neighborhood. They compare a baseline with one scenario of car-sharing. | Relative reduction with respect to the baseline. Assumption of reduction in the distance travelled due to special build conditions | Simulation of zero emission neighborhood | 10.1016/j.buildenv.2020.107528 |
| 3 | Bike sharing | Dockless shared bikes | Material depletion | Resource depletion | City | Asia | Raw materials<br>Production<br>Maintenance<br>Infrastructure (docks) | Comparison of three types of bike sharing systems: Stationary, free-floating and privately owned bike | They build the scenarios considering different amounts of shared bikes and usage rate. To extract the values I used the business as usual for the usage | Positive for both dockless and docked compared with privately owned. Between dockless and docked, dockless perform better. | 10.1016/j.jclepro.2020.124416 |
| 4 | Bike sharing | NA | Energy consumption | Resource depletion | Region | North America | Production (no raw material extraction)<br>Use<br>Maintenance<br>EoL | Comparison of two transport systems in a university campus: bus or bike sharing | | The bike system presents more environmental benefits than the bus system | 10.3390/su13010158 |
| 4 | Bike sharing | NA | $CO_2$ emissions | Climate impacts | Region | North America | Production<br>Use<br>Maintenance<br>EoL | Comparison of two transport systems in a university campus: bus or bike sharing | | The bike system presents more environmental benefits than the bus system | 10.3390/su13010158 |
| 5 | E-scooters | NA | $CO_2$ emissions | Climate impacts | City | Europé | Raw material<br>Production<br>Use<br>Maintenance<br>EoL | Assessment of emissions when e-scooters are used. The range is given by the different lifespans that the e-scooters might have in the shared system (min: 1058 km - max: 10000 km) | | The results depend on which transport mode the e-scooters replace. If they replace bikes or walking then there isn't any improvement. But if they replace cars there is an expected decrease in emissions. In the study they were found to replace walking and biking thus in this context they wouldn't represent gains. | 10.18757/ejtir.2020.20.4.4912 |
| 6 | E-scooters | NA | $CO_2$ emissions | Climate impacts | City | Europé | Production<br>Use<br>Maintenance<br>EoL<br>Infrastructure | Comparison of several urban transport modes | Conducted a survey to explore the modal shift in users of the e-scooters. They explore scenarios where the life time of the scooter is extended and then the impacts reduce considerably but not sufficiently. | Results show negative effects of e-scooter sharing because it replaces trips done by metro and walking. Emissions from e-scooters come from servicing and production. | 10.1016/j.jclepro.2020.122898 |
| 7 | Bike sharing | Dockless | $CO_2$ emissions | Climate impacts | Country | Asia | Production<br>Use<br>EoL | Evaluation of the emissions of bike sharing. This results assume that the bike is in the system for 686 days. | | For a bike to represent emission savings it needs to be used for 22 month. They assessed how the need for rebalancing will increase if the fleet of shared bikes increase and mention this as a key variable. Focus on disposal methods. | 10.1016/j.resconrec.2020.105011 |
| 8 | Car-sharing | B2C | GHG emissions | Climate impacts | Individual | USA<br>Europé | Production<br>Infrastructure<br>Use | Evaluation of the emissions of transport before and after car-sharing was introduced in three cities | | There is a decrease in all cities given that car-sharing users reduce private driving and increase vehicle occupancy | 10.1016/j.jclepro.2020.121869 |
| 9 | Bike sharing | Dockless | GHG emissions | Climate impacts | City | Asia | Manufacturing<br>Use<br>Maintenance (Rebalance) | Evaluation of the actual bike sharing system and three scenarios that assessed changes in the fleet size and the rebalancing strategy. | Scenario based with sensitivity analysis that focus on two variables rebalancing frequency and fleet size. | The rebalancing strategy was modelled in the scenarios, while the fleet size was modelled during the sensitivity analysis.<br>SC1 -Rebalance the system once a day<br>SC2 - Rebalance the system two times per day<br>SC3 - Rebalance the system three times per day<br>The scenario with lowest emissions is the one that is balanced once a day, too little or too big fleet increases the emissions. Inefficient rebalancing cannot be compensated by small fleets. | 10.1016/j.trc.2020.102705 |
| 10 | E-scooters | NA | GWP | Climate impacts | City | Europé | Production<br>Use | Comparison of scenarios where variables are changed. The baseline is compared with a modal shift scenario and extended lifetime in the shared system | The paper doesn't clarify what the base case represents. | The increase in the impacts is due to the short lifetime the e-scooters have in the shared system. The sensitivity analysis shows that the lifetime of the scooters is a very important variable to consider when assessing impacts. When comparing the personal scooter with a shared one the results show that personal scooters preform better due to a longer life time and better usage | 10.3390/su12051803 |
| 10 | E-scooters | NA | Fine particulate matter | Air quality | City | Europé | Production<br>Use | Comparison of scenarios where the baseline is considered. The modal shift and extended lifetime in the shared system | The paper doesn't clarify what the base case represents. | The increase in the impacts is due to the short lifetime the e-scooters have in the shared system. The sensitivity analysis shows that the lifetime of the scooters is a very important variable to consider when assessing impacts. When comparing the personal scooter with a shared one the results show that personal scooters preform better due to a longer life time and better usage | 10.3390/su12051803 |
| 10 | E-scooters | NA | Mineral resource scarcity | Resource depletion | City | Europé | Production<br>Use | Comparison of scenarios where the baseline is considered. The modal shift and extended lifetime in the shared system | The paper doesn't clarify what the base case represents. | The increase in the impacts is due to the short lifetime the e-scooters have in the shared system. The sensitivity analysis shows that the lifetime of the scooters is a very important variable to consider when assessing impacts. When comparing the personal scooter with a shared one the results show that personal scooters preform better due to a longer life time and better usage | 10.3390/su12051803 |
| 10 | E-scooters | NA | Fossil resource scarcity | Resource depletion | City | Europé | Production<br>Use | Comparison of scenarios where the baseline is considered. The modal shift and extended lifetime in the shared system | The paper doesn't clarify what the base case represents. | The increase in the impacts is due to the short lifetime the e-scooters have in the shared system. The sensitivity analysis shows that the lifetime of the scooters is a very important variable to consider when assessing impacts. When comparing the personal scooter with a shared one the results show that personal scooters preform better due to a longer life time and better usage | 10.3390/su12051803 |
| 11 | Bike sharing | Dockles and docked shared bikes | GHG emissions | Climate impacts | City | Other | Production<br>Use<br>EoL | Comparison of a smart bike sharing system both stationary and free-floating with private bikes. | They compare private, docked (smart docked) and dockless bikes (smart bike). | The docked and dockless bikes had a higher impact than private due to the electronics that are used. The dockless performed worse due to rebalancing. In order to present sustainability gains the shared bike must replace a certain amount of travelled km by car, if the bike sharing system is replacing private bike or walking then impacts are negative. | 10.1111/jiec.12860 |

| | | | | | | | | | | | |
|---|---|---|---|---|---|---|---|---|---|---|---|
| 12 | Car-sharing | Not specify | Energy use | Resource depletion | Country | Asia | Use | Comparison of before and after car-sharing was offered in the city (the car-sharing system has electric vehicles) | They used a survey to model the probability of the adoption of CS in China. After this they model the potential growth of the market and then calculated fuel savings, CO2 emissions and land use. | Gains relate with the fact that electrical vehicles were used to model | 10.1007/s11027-019-09893-2 |
| 12 | Car-sharing | Not specify | GHG emissions | Climate impacts | Country | Asia | Use | Comparison of before and after car-sharing was offered in the city (the car-sharing system has electric vehicles) | They used a survey to model the probability of the adoption of CS in China. After this they model the potential growth of the market and then calculated fuel savings, CO2 emissions and land use. | Gains relate with the fact that electrical vehicles were used to model | 10.1007/s11027-019-09893-2 |
| 12 | Car-sharing | Not specify | Land use | Land use | Country | Asia | Use | Comparison of before and after car-sharing was offered in the city (the car-sharing system has electric vehicles) | They used a survey to model the probability of the adoption of CS in China. After this they model the potential growth of the market and then calculated fuel savings, CO2 emissions and land use. | Gains relate with the fact that electrical vehicles were used to model | 10.1007/s11027-019-09893-2 |
| 13 | Car-sharing | Fre- floting<br>Stationary based | GWP | Climate impacts | City | Asia | Production<br>Use | Comparison of different types of car-sharing accounting for one year passenger transport. These compared to private driving. | They consider different business models and assign different lifetimes to the vehicles depending on the business model. | If any form of car sharing replaces private cars then they assume a reduction in the emissions. However, if impacts are looked without considering changes in travelling behavior then the impacts can be higher than the savings. | 10.1016/j.scitotenv.2019.06.111 |
| 14 | Carpooling | Carpooling<br>Car-sgaring | Eco points | Indicator | Individual | Latin America | Use | Comparison of travelling to work by yourself, with one other person or other two people | The paper models the mobility portfolio of individuals. Considering commuting, shopping, vacation and other types of trip types. Interesting for the literature review are the carpooling scenarios | | 10.1016/j.jclepro.2019.06.203 |
| 15 | E-scooters | NA | GWP | Climate impacts | Business model | North America | Production<br>Use<br>EoL | Comparison of emissions of e-scooters with other transport systems. | Results that show how the e-scooter sharing compares with other transport modes | It depends on which transport modes it replaces, if it replaces car. If it replaces bikes, bus with high ridership or electric bikes it doesn't decreases the impacts | 10.1088/1748-9326/ab2da8 |
| 15 | E-scooters | NA | GWP | Climate impacts | Business model | North America | Production<br>Use<br>EoL | Comparison of emissions of e-scooters with other transport systems. They included the evaluation of the e-scooter sharing system considering changes in certain variables: low collection distance, battery depletion limit, high vehicle efficiency and high scooter lifetime. | After the LCA a Monte Carlo simulation was performed in order to explore which variables are most sensitive in the model. It shows that the most sensitive is lifetime in the shared system , frequency of use of the scooter and rebalancing | | 10.1088/1748-9326/ab2da8 |
| 15 | E-scooters | NA | Acidification | Ecosystem damage | Business model | North America | Production<br>Use<br>EoL | Comparison of emissions of e-scooters with other transport systems. They included the evaluation of the e-scooter sharing system considering changes in certain variables: low collection distance, battery depletion limit, high vehicle efficiency and high scooter lifetime. | After the LCA a Monte Carlo simulation was performed in order to explore which variables are most sensitive in the model. It shows that the most sensitive is lifetime in the shared system , frequency of use of the scooter and rebalancing | | 10.1088/1748-9326/ab2da8 |
| 15 | E-scooters | NA | Eutrophication | Ecosystem damage | Business model | North America | Production<br>Use<br>EoL | Comparison of emissions of e-scooters with other transport systems. They included the evaluation of the e-scooter sharing system considering changes in certain variables: low collection distance, battery depletion limit, high vehicle efficiency and high scooter lifetime. | After the LCA a Monte Carlo simulation was performed in order to explore which variables are most sensitive in the model. It shows that the most sensitive is lifetime in the shared system , frequency of use of the scooter and rebalancing | | 10.1088/1748-9326/ab2da8 |
| 16 | Bike sharing | Dockless and docked | Total normalized environmental impacts | Indicator | City | North America | Production (Bike and dock)<br>Use<br>EoL | Comparison of two bike sharing systems, stationary and free-floating, with other modes of transport | | Total normalized indicator: Dockless bikes perform better | 10.1016/j.resconrec.2019.03.003 |
| 16 | Bike sharing | Dockless and docked | GHG | Climate impacts | City | North America | Production (Bike and dock)<br>Use<br>EoL | Comparison of two bike sharing systems, stationary and free-floating, with other modes of transport | | GHG: To have positive effects the docked bikes need to replace 7% of vehicle trips whiles dockless needs to replace 34%. Comparing docked and dockless bikes the docked ones perform better. Additionally, emissions depend on the rebalancing strategy and on the number of bikes in the system | 10.1016/j.resconrec.2019.03.003 |
| 17 | Carpooling | NA | Energy consumption | Resource depletion | City | Asia | Production<br>Use | Assessment of the environmental savings due to carpooling differentiating three cities. | This articles uses data from Didi when it offers the service of carpooling. This article includes the results of 10.1016/j.apenergy.2017.01.052. Mixing of LCA and IO. | | 10.3390/en11113214 |
| 17 | Carpooling | NA | CO2 emissions | Climate impacts | City | Asia | Production<br>Use | Assessment of the environmental savings due to carpooling differentiating three cities. | This articles uses data from Didi when it offers the service of carpooling. This article includes the results of 10.1016/j.apenergy.2017.01.052. Mixing of LCA and IO. | | 10.3390/en11113214 |
| 17 | Carpooling | NA | Primary PM2.5 | Air quality | City | Asia | Production<br>Use | Assessment of the environmental savings due to carpooling differentiating three cities. | This articles uses data from Didi when it offers the service of carpooling. This article includes the results of 10.1016/j.apenergy.2017.01.052. Mixing of LCA and IO. | | 10.3390/en11113214 |
| 17 | Carpooling | NA | SO2 emissions | Air quality | City | Asia | Production<br>Use | Assessment of the environmental savings due to carpooling differentiating three cities. | This articles uses data from Didi when it offers the service of carpooling. This article includes the results of 10.1016/j.apenergy.2017.01.052. Mixing of LCA and IO. | | 10.3390/en11113214 |
| 17 | Carpooling | NA | NOx emissions | Air quality | City | Asia | Production<br>Use | Assessment of the environmental savings due to carpooling differentiating three cities. | This articles uses data from Didi when it offers the service of carpooling. This article includes the results of 10.1016/j.apenergy.2017.01.052. Mixing of LCA and IO. | | 10.3390/en11113214 |
| 18 | Bike sharing | Dockless | Fuel use | Resource depletion | City | Asia | Use (Modal shift) | Assessment of savings in a year after bike sharing was introduced. | The investigate the length of the trips by bike and then modelled energy savings for trips who's lengths was more than 1 km. They assume that these trips were done by taxi. Thus they compare taxi driving and bike sharing. | Not surprising that the results show a decrease. The model excludes impacts from rebalancing | 10.1016/j.apenergy.2018.03.101 |
| 18 | Bike sharing | Dockless | CO2 emissions | Climate impacts | City | Asia | Use (Modal shift) | Assessment of savings in a year after bike sharing was introduced. | The investigate the length of the trips by bike and then modelled energy savings for trips who's lengths was more than 1 km. They assume that these trips were done by taxi. Thus they compare taxi driving and bike sharing. | Not surprising that the results show a decrease. The model excludes impacts from rebalancing | 10.1016/j.apenergy.2018.03.101 |

| | | | | | | | | | | | |
|---|---|---|---|---|---|---|---|---|---|---|---|
| 18 | Bike sharing | Dockless | NOx emissions | Air quality | City | Asia | Use (Modal shift) | Assessment of savings in a year after bike sharing was introduced. | The investigate the length of the trips by bike and then modelled energy savings for trips who's lengths was more than 1 km. They assume that these trips were done by taxi. Thus they compare taxi driving and bike sharing. | Not surprising that the results show a decrease. The model excludes impacts from rebalancing | 10.1016/j.apenergy.2018.03.101 |
| 19 | Carpooling | NA | Energy consumption | Resource depletion | City | Asia | Production<br>Use | Assessment of the environmental savings due to carpooling in one city. | This articles uses data from Didi when it offers the service of carpooling. This article includes the results of 10.3390/en11113214. In addition to this data, they applied a survey where they ask users changes in modal shift and ownership. Changes in modal shift were modelled in the LCA and changes in ownership were modelled with IO | Changes from modal shift and car ownership are present in the reduction of the emissions | 10.1016/j.apenergy.2017.01.052 |
| 19 | Carpooling | NA | CO2 emissions | Climate impacts | City | Asia | Production<br>Use | Assessment of the environmental savings due to carpooling in one city. | This articles uses data from Didi when it offers the service of carpooling. This article includes the results of 10.3390/en11113214. In addition to this data, they applied a survey where they ask users changes in modal shift and ownership. Changes in modal shift were modelled in the LCA and changes in ownership were modelled with IO | Changes from modal shift and car ownership are present in the reduction of the emissions | 10.1016/j.apenergy.2017.01.052 |
| 19 | Carpooling | NA | Primary PM2.5 | Air quality | City | Asia | Production<br>Use | Assessment of the environmental savings due to carpooling in one city. | This articles uses data from Didi when it offers the service of carpooling. This article includes the results of 10.3390/en11113214. In addition to this data, they applied a survey where they ask users changes in modal shift and ownership. Changes in modal shift were modelled in the LCA and changes in ownership were modelled with IO | Changes from modal shift and car ownership are present in the reduction of the emissions | 10.1016/j.apenergy.2017.01.052 |
| 19 | Carpooling | NA | SO2 emissions | Air quality | City | Asia | Production<br>Use | Assessment of the environmental savings due to carpooling in one city. | This articles uses data from Didi when it offers the service of carpooling. This article includes the results of 10.3390/en11113214. In addition to this data, they applied a survey where they ask users changes in modal shift and ownership. Changes in modal shift were modelled in the LCA and changes in ownership were modelled with IO | Changes from modal shift and car ownership are present in the reduction of the emissions | 10.1016/j.apenergy.2017.01.052 |
| 19 | Carpooling | NA | NOx emissions | Air quality | City | Asia | Production<br>Use | Assessment of the environmental savings due to carpooling in one city. | This articles uses data from Didi when it offers the service of carpooling. This article includes the results of 10.3390/en11113214. In addition to this data, they applied a survey where they ask users changes in modal shift and ownership. Changes in modal shift were modelled in the LCA and changes in ownership were modelled with IO | Changes from modal shift and car ownership are present in the reduction of the emissions | 10.1016/j.apenergy.2017.01.052 |
| 20 | Car-sharing | NA | GHG emissions | Climate impacts | Country | North America | Manufacturing<br>Use<br>EoL<br>Infrastructure | Comparison of before and after car-sharing was introduced considering three scenarios of usage: low, medium and high. | To model changes in environmental impacts they explore changes in vehicle ownership, VKT, parking infrastructure and modal shift. All these assumptions were taken from other studies and they build three scenarios (low, middle and high) | Most of the environmental gains come from changes in ways of travel. However, gains are sensitive to occupancy of PT | 10.1016/j.trd.2016.05.012 |
| 20 | Car-sharing | NA | Energy use | Resource depletion | Country | North America | Manufacturing<br>Use<br>EoL<br>Infrastructure | Comparison of before and after car-sharing was introduced considering three scenarios of usage, low, medium and high. | To model changes in environmental impacts they explore changes in vehicle ownership, VKT, parking infrastructure and modal shift. All these assumptions were taken from other studies and they build three scenarios (low, middle and high) | Most of the environmental gains come from changes in ways of travel. However, gains are sensitive to occupancy of PT | 10.1016/j.trd.2016.05.012 |
| 21 | Car-sharing | NA | GHG emissions | Climate impacts | City | North America | Use (tail pipe) | Comparison of before and after car-sharing was introduced using assumptions to build the scenarios | They classify the households in three different categories and explored car ownership. They model modal shift, newer cars in the shared fleet, vehicle optimization factor, trip aggregation. | The households that have the highest potential of saving emissions are the households with children. The most savings come from the modal shift. In the sensitivity analysis if the trip is done by more efficient vehicles there are more saving but if the modal shift happens to private cars to public transport the possible emission reduction is less | 10.1088/1748-9326/10/12/124017 |
| 22 | Car-sharing | NA | GHG emissions | Climate impacts | Household | North America | Manufacturing<br>Use<br>EoL<br>Infrastructure | Comparison of before and after car-sharing was introduced using results from a survey | They measure direct impacts (due to changes in travelling behavior ) and full impact (forego a vehicle). Direct impacts have a low level of uncertainty and full impacts a higher uncertainty. This was calculated considering results from a survey. The main metric is changes in VKT in the households | Users that gain access to a car increase their emissions. Users that forego a car decrease the. If this is quantified at a total level then there are environmental gains. | 10.1109/TITS.2011.2158539 |
| 23 | Car-sharing | Free- floating | GHG emissions | Climate impacts | City | Europé | Use | Comparison of before and after car-sharing was introduced. | They conducted a survey to pedestrians. Based on this information they explored how they would change they travelling habits if the got engaged in CS. | Free-floating car-sharing - They calculated reduction in car ownership | 10.1016/j.ecolecon.2011.03.014 |
| 24 | Carpooling | NA | CO2 emissions | Climate impacts | City | Europé | Use | Assessment of emission savings if people shifts from driving alone to car-pooling | Based on the national census they find the individuals that can potentially share their car. Thus there is a reduction in total kilometers travelled per year | | 10.3141/2163-11 |
| 25 | Bike sharing | Dockless | ADP Water | Resource depletion | City | Asia | Manufacturing<br>Use<br>EoL | Comparison of impacts of different transport modes. In the case of bike sharing two utility cases were considered: low and high. | | | 10.1016/j.spc.2021.02.008 |
| 25 | Bike sharing | Dockless | ADP Mineral and fossil | Resource depletion | City | Asia | Manufacturing<br>Use<br>EoL | Comparison of impacts of different transport modes. In the case of bike sharing two utility cases were considered: low and high. | | | 10.1016/j.spc.2021.02.008 |
| 25 | Bike sharing | Dockless | GWP | Climate impacts | City | Asia | Manufacturing<br>Use<br>EoL | Comparison of impacts of different transport modes. In the case of bike sharing two utility cases were considered: low and high. | There is a decrease in emissions given that bike sharing replaces more pollutant transport modes. | | 10.1016/j.spc.2021.02.008 |
| 26 | Carpooling | Ridesharing, carpooling and car-sharing | GWP | Climate impacts | City | Asia - North America | Manufacturing<br>Use<br>EoL | Comparison of impacts in different transport systems. Toronto | They assessed the impact of different shared mobility modes in two different cities. It's interesting to see that depending on the city characteristics results vary | Carpooling has more potential than car-sharing | 10.1016/j.scitotenv.2021.145014 |
| 26 | Bike sharing | Dockless and docked | GWP | Climate impacts | City | Asia - North America | Manufacturing<br>Use<br>EoL | Comparison of impacts in different transport systems. Beijing | They assessed the impact of different shared mobility modes in two different cities. It's interesting to see that depending on the city characteristics results vary | Compared to private bikes shared bikes this due to the rebalancing and due to the station and dock lifecycle | 10.1016/j.scitotenv.2021.145014 |

| | | | | | | | | | | | |
|---|---|---|---|---|---|---|---|---|---|---|---|
| 26 | Bike sharing | Dockless and docked | GWP | Climate impacts | City | Asia - North America | Manufacturing<br>Use<br>EoL | Comparison of impacts in different transport systems. Toronto | They assessed the impact of different shared mobility modes in two different cities. It's interesting to see that depending on the city characteristics results vary | Compared to private bikes shared bikes this due to the rebalancing and due to the station and dock lifecycle | 10.1016/j.scitotenv.2021.145014 |
| 27 | Bike sharing | Dockless bikes | GHG emissions | Climate impacts | City | Asia | Production<br>Use | Comparison of impacts before and after bike sharing was implemented in a city | They have a big dataset from where they can extract the distance and duration of the trips. They run a probabilistic model to determine the probability of the traveler taking other transport mode and calculate savings in emissions. In addition, they have a regression analysis to link the characteristics of the city with the sustainability outcomes of the sharing system. | The trips that could have been done by walking have a negative impact on the environment, while other trips that replace GHG emission intensive trips are beneficial. | 10.1016/j.jclepro.2021.126423 |
| 28 | Car-sharing | NA | Energy Used | Resource depletion | City | Asia | Production<br>Use | Evaluation of energy savings of car-sharing in a specific geographical area | They compared the environmental impacts of shared vehicles with ICV or EV. Also quantify the benefits that sharing EV can bring to the system when changes in travelling behavior occurred due to sharing | | 10.1016/j.est.2021.102334 |
| 29 | Micro-mobili | NA | Climate impacts | Climate impacts | Business model | Europé | Production<br>Use(Maintenance)<br>Infrastructure<br>EoL | Comparison of transport modes per pkt | They compared sharing micro mobility options vs private ownership. The system boundaries are wide but a disadvantage that I see is that they didn't include changes in user behavior or the modal shift due to sharing | Each transport mode has different performance. Private bike performs better than the shared, because of the dock stations in all categories. While there are trade-off in the rest of the micro mobility modes | 10.1016/j.trd.2021.102743 |
| 29 | Micro-mobili | NA | Resource depletion | Resource depletion | Business model | Europé | Production<br>Use(Maintenance)<br>Infrastructure<br>EoL | Comparison of transport modes per pkt | They compared sharing micro mobility options vs private ownership. The system boundaries are wide but a disadvantage that I see is that they didn't include changes in user behavior or the modal shift due to sharing | Each transport mode has different performance. Private bike performs better than the shared, because of the dock stations in all categories. While there are trade-off in the rest of the micro mobility modes | 10.1016/j.trd.2021.102743 |
| 29 | Micro-mobili | NA | Ecosystem damage | Ecosystem damage | Business model | Europé | Production<br>Use(Maintenance)<br>Infrastructure<br>EoL | Comparison of transport modes per pkt | They compared sharing micro mobility options vs private ownership. The system boundaries are wide but a disadvantage that I see is that they didn't include changes in user behavior or the modal shift due to sharing | Each transport mode has different performance. Private bike performs better than the shared, because of the dock stations in all categories. While there are trade-off in the rest of the micro mobility modes | 10.1016/j.trd.2021.102743 |
| 30 | Car-sharing | p2p and B2C | Climate impacts | Climate impacts | City/Individual | Europé | Use | Comparison of transport emissions considering changes in travelling behavior after engaging with car-sharing | Focus on the use phase, including emissions from fuel production and tail pipe | If individuals gain access to a vehicle there is an increase in the emissions. If people foregoes their vehicle there is a decrease. At a city level total effects depend on how individuals change their travelling habits | https://doi.org/10.3390/su13042418 |
| 31 | Car-sharing | Stationary base | CH4 | Climate impacts | City | Europé | Use | Comparison of private diving with car-sharing | They conducted a survey to the users of car sharing in Palermo and their findings point out to the fact that car sharing replaces the purchase of a second vehicle and that the modal share changes. They report they travel the same amount of km. After they took this information and calculated emissions saved considering the characteristics of the average car and the car from CS. | The cars in the car sharing fleet present better performance than private owned vehicles. There was a decrease of emissions but this decrease is limited. | https://doi.org/10.1016/j.trpro.2020.08.271 |
| 31 | Car-sharing | Stationary base | CO | Climate impacts | City | Europé | Use | Comparison of private diving with car-sharing | They conducted a survey to the users of car sharing in Palermo and their findings point out to the fact that car sharing replaces the purchase of a second vehicle and that the modal share changes. They report they travel the same amount of km. After they took this information and calculated emissions saved considering the characteristics of the average car and the car from CS. | The cars in the car sharing fleet present better performance than private owned vehicles. There was a decrease of emissions but this decrease is limited. | https://doi.org/10.1016/j.trpro.2020.08.271 |
| 31 | Car-sharing | Stationary base | CO2 emissions | Climate impacts | City | Europé | Use | Comparison of private diving with car-sharing | They conducted a survey to the users of car sharing in Palermo and their findings point out to the fact that car sharing replaces the purchase of a second vehicle and that the modal share changes. They report they travel the same amount of km. After they took this information and calculated emissions saved considering the characteristics of the average car and the car from CS. | The cars in the car sharing fleet present better performance than private owned vehicles. There was a decrease of emissions but this decrease is limited. | https://doi.org/10.1016/j.trpro.2020.08.271 |
| 31 | Car-sharing | Stationary base | NOx emissions | Air quality | City | Europé | Use | Comparison of private diving with car-sharing | They conducted a survey to the users of car sharing in Palermo and their findings point out to the fact that car sharing replaces the purchase of a second vehicle and that the modal share changes. They report they travel the same amount of km. After they took this information and calculated emissions saved considering the characteristics of the average car and the car from CS. | The cars in the car sharing fleet present better performance than private owned vehicles. There was a decrease of emissions but this decrease is limited. | https://doi.org/10.1016/j.trpro.2020.08.271 |
| 31 | Car-sharing | Stationary base | Ozone depletion | Ozone depletion | City | Europé | Use | Comparison of private diving with car-sharing | They conducted a survey to the users of car sharing in Palermo and their findings point out to the fact that car sharing replaces the purchase of a second vehicle and that the modal share changes. They report they travel the same amount of km. After they took this information and calculated emissions saved considering the characteristics of the average car and the car from CS. | The cars in the car sharing fleet present better performance than private owned vehicles. There was a decrease of emissions but this decrease is limited. | https://doi.org/10.1016/j.trpro.2020.08.271 |
| 31 | Car-sharing | Stationary base | PM10 | Air quality | City | Europé | Use | Comparison of private diving with car-sharing | They conducted a survey to the users of car sharing in Palermo and their findings point out to the fact that car sharing replaces the purchase of a second vehicle and that the modal share changes. They report they travel the same amount of km. After they took this information and calculated emissions saved considering the characteristics of the average car and the car from CS. | The cars in the car sharing fleet present better performance than private owned vehicles. There was a decrease of emissions but this decrease is limited. | https://doi.org/10.1016/j.trpro.2020.08.271 |
| 32 | Car-sharing | Stationary base | Climate impacts | Climate impacts | City | Europé | Use | Comparison of before and after car-sharing was introduced in a city during one year | They build scenarios for car-sharing where they consider the speed, the type of driving cycle and other variables. They calculated WTW emissions. In the simulations they include different type of vehicles to see how this would improve the system. | As the fleet is today there are environmental benefits from car sharing. However, these benefits increase considerably when the fleet changes to hybrid or to electric. | https://doi.org/10.1016/j.sbspro.2014.01.035 |

| | | | | | | | | | | | |
|---|---|---|---|---|---|---|---|---|---|---|---|
| 32 | Car-sharing | Stationary base | Resource depletion | Resource depletion | City | Europé | Use | Comparison of before and after car-sharing was introduced in a city during one year | They build scenarios for car-sharing where they consider the speed, the type of driving cycle and other variables. They calculated WTW emissions. In the simulations they include different type of vehicles to see how this would improve the system. | As the fleet is today there are environmental benefits from car sharing. However, these benefits increase considerably when the fleet changes to hybrid or to electric. | https://doi.org/10.1016/j.sbspro.2014.01.035 |
| 33 | Carpooling | NA | CO2 emissions | Climate impacts | Region | Europé | Use | Comparison before and after carpooling. Considering an increase in vehicle occupancy in Scotland. | They evaluate several transport mechanisms of ICT in emissions. The first strategy is reduction in travelled time and the next one is the increase of vehicle occupancy. The region evaluated is Scotland and everything is based on modelling scenarios | | 10.1260/2046-0430.4.3.277 |
| 34 | Carpooling | NA | CO2 emissions | Climate impacts | Region | Europé | Use | Comparison of before and after carpooling was available. | The study is a project to reduce CO2 emissions and improve local air quality by the integration of mobility. The project is focused on sharing knowledge and building capacity between different regions in order to learn from each other. In this case the paper is based on an industrial region in Italy where they identified improvement opportunities. | | 10.2495/SC130882 |
| 35 | Carpooling | NA | CO2 emissions | Climate impacts | Country | Europé | Use | Assessment of three carpooling scenarios. SC1 - Carpooling 5 days a week, SC2 - Carpooling 4 days a week and SC3 - Carpooling 3 days a week. | The article is based on the results from the census in Ireland. Here people described if they were car sharing users and as a result scenarios were built where emission savings were estimated. | | 10.1016/j.trd.2009.07.008 |
| 36 | Bike sharing | Stationary base | CO2 emissions | Climate impacts | City | Europé | Manufacture<br>Use<br>EoL | Comparison of the bike sharing system with travelling by bus. The assessments includes a perspective of 5 yrs. | Very extensive LCA that even includes impacts from diet and breathing. They also include impacts from transportation of spare parts and the manufactures e-scooters | | 10.1016/j.scs.2021.103012 |
| 37 | E-moped | Dockless | CO2 emissions | Climate impacts | City | Europé | Manufacture<br>Use<br>EoL | Assessment of e-moped system considering three different sizes (2500, 10000 and 50000) and energy sources (current and renewable). | The study explores using an agent base the probability that e-scooters will replace trips done by private cars. They found that depending on the fleet size the percentage of shared cars that can be replaced varies. Larger fleets lead to more replacement but to more impacts from production and less from rebalancing. | Small size fleets and renewable energy have the lowest impacts | 10.3390/wevj12030096 |
| 37 | E-moped | Dockless | SO2 emissions | Air quality | City | Europé | Manufacture<br>Use<br>EoL | Assessment of e-moped system considering three different sizes (2500, 10000 and 50000) and energy sources (current and renewable). | The study explores using an agent base the probability that e-scooters will replace trips done by private cars. They found that depending on the fleet size the percentage of shared cars that can be replaced varies. Larger fleets lead to more replacement but to more impacts from production and less from rebalancing. | Small size fleets and renewable energy have the lowest impacts | 10.3390/wevj12030096 |
| 37 | E-moped | Dockless | Eutrophication | Ecosystem damage | City | Europé | Manufacture<br>Use<br>EoL | Assessment of e-moped system considering three different sizes (2500, 10000 and 50000) and energy sources (current and renewable). | The study explores using an agent base the probability that e-scooters will replace trips done by private cars. They found that depending on the fleet size the percentage of shared cars that can be replaced varies. Larger fleets lead to more replacement but to more impacts from production and less from rebalancing. | Small size fleets and renewable energy have the lowest impacts | 10.3390/wevj12030096 |
| 37 | E-moped | Dockless | PM 2.5 | Air quality | City | Europé | Manufacture<br>Use<br>EoL | Assessment of e-moped system considering three different sizes (2500, 10000 and 50000) and energy sources (current and renewable). | The study explores using an agent base the probability that e-scooters will replace trips done by private cars. They found that depending on the fleet size the percentage of shared cars that can be replaced varies. Larger fleets lead to more replacement but to more impacts from production and less from rebalancing. | Small size fleets and renewable energy have the lowest impacts | 10.3390/wevj12030096 |
| 37 | E-moped | Dockless | Resource depletion | Resource depletion | City | Europé | Manufacture<br>Use<br>EoL | Assessment of e-moped system considering three different sizes (2500, 10000 and 50000) and energy sources (current and renewable). | The study explores using an agent base the probability that e-scooters will replace trips done by private cars. They found that depending on the fleet size the percentage of shared cars that can be replaced varies. Larger fleets lead to more replacement but to more impacts from production and less from rebalancing. | Small size fleets and renewable energy have the lowest impacts | 10.3390/wevj12030096 |
| 38 | Car-sharing | Car-sharing/Ridesharing | Several impacts | na | Country | Europé | Manufacture<br>Use<br>EoL | Evaluation of the impacts of electrification of the fleet in the UK. An additional scenario was developed where sharing was considered as an alternative | This study focus on the electrification of the fleet in UK, then a second scenario is build to assess impacts of both electrification and sharing over time. Extracting data from this paper was not possible because they present a graph but the number that this graph represent are not specified. | Observing the general trend of the graphs not surprisingly there is a decrease in impacts if people shared their vehicles. | 10.1016/j.resconrec.2021.105818 |
| 39 | E-moped | NA | CO2 emissions | Climate impacts | Business model | Europé | Manufacturing<br>Use<br>EoL | Comparison of the e-moped emissions with other transport modes. Additionally, they assessed variables in the e-moped system and tested their impacts in emission | Several improvements to the actual system are modeled that include changes in rebalancing, lifetime or energy source | | 10.3390/su13158297 |
| 40 | Carpooling | NA | CO2 emissions | Climate impacts | Neighborhood | Europé | Use | Evaluation of different mobility scenarios that include the increase of vehicle occupancy in a given neighborhood. This estimations account for projections up to 2060 | Evaluation of different climate strategies for example use of shared mobility and increased use of public transport, extended lifetimes and local energy production | | 10.1007/s11367-021-01973-3 |
| 40 | Carpooling | NA | Freshwater Eutrophication | Ecosystem damage | Neighborhood | Europé | Use | Evaluation of different mobility scenarios that include the increase of vehicle occupancy in a given neighborhood. This estimations account for projections up to 2061 | Evaluation of different climate strategies for example use of shared mobility and increased use of public transport, extended lifetimes and local energy production | | 10.1007/s11367-021-01973-3 |
| 40 | Carpooling | NA | Material depletion | Resource depletion | Neighborhood | Europé | Use | Evaluation of different mobility scenarios that include the increase of vehicle occupancy in a given neighborhood. This estimations account for projections up to 2062 | Evaluation of different climate strategies for example use of shared mobility and increased use of public transport, extended lifetimes and local energy production | | 10.1007/s11367-021-01973-3 |
| 40 | Carpooling | NA | Terrestrial acidification | Ecosystem damage | Neighborhood | Europé | Use | Evaluation of different mobility scenarios that include the increase of vehicle occupancy in a given neighborhood. This estimations account for projections up to 2063 | Evaluation of different climate strategies for example use of shared mobility and increased use of public transport, extended lifetimes and local energy production | | 10.1007/s11367-021-01973-3 |
